# Influence of obesity-related risk factors in the aetiology of glioma

**DOI:** 10.1038/s41416-018-0009-x

**Published:** 2018-03-13

**Authors:** Linden Disney-Hogg, Amit Sud, Philip J. Law, Alex J. Cornish, Ben Kinnersley, Quinn T. Ostrom, Karim Labreche, Jeanette E. Eckel-Passow, Georgina N. Armstrong, Elizabeth B. Claus, Dora Il’yasova, Joellen Schildkraut, Jill S. Barnholtz-Sloan, Sara H. Olson, Jonine L. Bernstein, Rose K. Lai, Anthony J. Swerdlow, Matthias Simon, Per Hoffmann, Markus M. Nöthen, Karl-Heinz Jöckel, Stephen Chanock, Preetha Rajaraman, Christoffer Johansen, Robert B. Jenkins, Beatrice S. Melin, Margaret R. Wrensch, Marc Sanson, Melissa L. Bondy, Richard S. Houlston

**Affiliations:** 1 Division of Genetics and Epidemiology, The Institute of Cancer Research, London, SW7 3RP UK; 2 Case Comprehensive Cancer Center, School of Medicine, Case Western Reserve University, Cleveland, OH 44106 USA; 3 Division of Biomedical Statistics and Informatics, Mayo Clinic College of Medicine, Rochester, MI 55905 USA; 4 Section of Epidemiology and Population Sciences, Department of Medicine, Dan L. Duncan Comprehensive Cancer Center, Baylor College of Medicine, Houston, TX 77030 USA; 5 School of Public Health, Yale University, New Haven, CT 06510 USA; 6 Department of Neurosurgery, Brigham and Women’s Hospital, Boston, MA 02115 USA; 7 Department of Epidemiology and Biostatistics, School of Public Health, Georgia State University, Atlanta, GA 30303 USA; 8 Duke Cancer Institute, Duke University Medical Center, Durham, NC 27710 USA; 9 Cancer Control and Prevention Program, Department of Community and Family Medicine, Duke University Medical Center, Durham, NC 27710 USA; 10 Department of Epidemiology and Biostatistics, Memorial Sloan Kettering Cancer Center, New York, NY 10017 USA; 11 Departments of Neurology and Preventive Medicine, Keck School of Medicine, University of Southern California, Los Angeles, CA 90033 USA; 12 Division of Breast Cancer Research, The Institute of Cancer Research, London, SW7 3RP UK; 13 Department of Neurosurgery, University of Bonn Medical Center, Sigmund-Freud-Str. 25, Bonn, 53105 Germany; 14 Human Genomics Research Group, Department of Biomedicine, University of Basel, Basel, 4031 Switzerland; 15 Department of Genomics, Life & Brain Center, University of Bonn, Bonn, 53127 Germany; 16 Institute of Human Genetics, University of Bonn School of Medicine and University Hospital Bonn, Bonn, 53127 Germany; 17 Institute for Medical Informatics, Biometry and Epidemiology, University Hospital Essen, University of Duisburg-Essen, Essen, 45147 Germany; 18 Division of Cancer Epidemiology and Genetics, National Cancer Institute, Bethesda, MD 20892 USA; 19 Danish Cancer Society Research Center, Survivorship, Danish Cancer Society, Copenhagen, 2100 Denmark; 20 Oncology Clinic, Finsen Centre, Rigshospitalet, University of Copenhagen, Copenhagen, 2100 Denmark; 21 Department of Laboratory Medicine and Pathology, Mayo Clinic Comprehensive Cancer Center, Mayo Clinic, Rochester, MI 55905 USA; 22 Department of Radiation Sciences, Umeå University, Umeå, 901 87 Sweden; 23 Department of Neurological Surgery, School of Medicine, University of California, San Francisco, CA 94143 USA; 24 Institute of Human Genetics, University of California, San Franciso, CA 94143 USA; 25 Sorbonne Universités UPMC Univ Paris 06 INSERM CNRS, U1127, UMR 7225, ICM, Paris, 75013 France; 26 AP-HP, Groupe Hospitalier Pitié-Salpêtrière, Service de Neurologie 2-Mazarin, Paris, 75013 France; 27 Division of Molecular Pathology, The Institute of Cancer Research, London, SW7 3RP UK

**Keywords:** Cancer genetics, Cancer epidemiology

## Abstract

**Background:**

Obesity and related factors have been implicated as possible aetiological factors for the development of glioma in epidemiological observation studies. We used genetic markers in a Mendelian randomisation framework to examine whether obesity-related traits influence glioma risk. This methodology reduces bias from confounding and is not affected by reverse causation.

**Methods:**

Genetic instruments were identified for 10 key obesity-related risk factors, and their association with glioma risk was evaluated using data from a genome-wide association study of 12,488 glioma patients and 18,169 controls. The estimated odds ratio of glioma associated with each of the genetically defined obesity-related traits was used to infer evidence for a causal relationship.

**Results:**

No convincing association with glioma risk was seen for genetic instruments for body mass index, waist-to-hip ratio, lipids, type-2 diabetes, hyperglycaemia or insulin resistance. Similarly, we found no evidence to support a relationship between obesity-related traits with subtypes of glioma–glioblastoma (GBM) or non-GBM tumours.

**Conclusions:**

This study provides no evidence to implicate obesity-related factors as causes of glioma.

## Introduction

Glioma is the most common primary intracranial tumour, accounting for around 80% of all malignant brain tumours.^1^ Thus far, few established risk factors for the development of glioma have been robustly identified.^2^

Obesity-related factors are increasingly being recognised as risk determinants for the development many of common cancers, such as those of the breast and colorectum.^3^ Evidence from epidemiological observational studies, for obesity-related traits being a risk factor for the development of glioma have, however been inconsistent, with only a subset of studies reporting a significant association.^4–9^ Furthermore, in contrast to most cancers, some studies have reported diabetes to be protective against glioma.^10–13^ Obesity-related exposures are however inherently interrelated,^14, 15^ and in traditional epidemiological studies it can be problematic to isolate specific risk factors that may exert a causal influence on disease from those that are merely associated with an underlying causal factor (i.e. confounded). In addition, findings can be affected by reverse causation.

Mendelian randomisation (MR) is an analytical approach to the traditional epidemiological study whereby genetic markers are used as proxies or instrumental variables (IVs) of environmental and lifestyle-related risk factors.^16^ Such genetic markers cannot be influenced by reverse causation and can act as unconfounded markers of exposures provided the variants are not associated with the disease through an alternative mechanism.^16^ Under these circumstances, the association between a genetic variant (or set of variants) and outcome of interest implies a causal relationship between the risk factor and outcome. MR has therefore been compared to a natural randomised controlled trial, circumventing some of the limitations of epidemiological observational studies.^17^ However, as IVs used in MR often explain a small proportion of the exposure phenotypic variance, large sample sizes are required to have sufficient power.^18^

To gain insight into the aetiology of glioma, we have examined the role of obesity-related risk factors in glioma using an MR-based framework. Specifically, we identified genetic variants associated with 10 key obesity-related risk factors from external genetic association studies. We implemented two-sample MR^19^ to estimate associations between these genetic variants with glioma risk using genome-wide association study (GWAS) data from the Glioma International Case-Control Consortium study (GICC).^20^

## Materials and methods

Two-sample MR was undertaken using GWAS data. Ethical approval was not sought for this specific project because all data came from the summary statistics of published GWAS, and no individual-level data were used.

### Genetic instruments for obesity and related risk factors

Genetic instruments were identified as a panel of single-nucleotide polymorphisms (SNPs) identified from recent meta-analyses or largest studies published to date. Specifically: (i) SNPs for body mass index (BMI) and waist-to-hip ratio (WHR) were identified from the Genetic Investigation of ANthropometric Traits (GIANT) consortium;^21, 22^ (ii) SNPs for circulating high-density and low-density lipoprotein cholesterol (HDL and LDL), total cholesterol and triglycerides, were identified from the Global Lipids Genetic Consortium (GLGC);^23^ (iii) SNPs for factors related to hyperglycaemia and hyperinsulinemia—fasting glucose, fasting insulin and 2-h post-challenge glucose, were obtained from the Meta-Analysis of Glucose and Insulin related traits Consortium (MAGIC)^24^ and (iv) SNPs for type-2 diabetes were identified from.^25^ For each SNP, we recovered the chromosome position, the effect estimate expressed in standard deviations (SD) of the trait per-allele along with the corresponding standard error (Supplementary Table 1). We restricted our analysis to SNPs associated at genome-wide significance (i.e. *P* ≤ 5.0 × 10^−8^) in individuals with European ancestry. To avoid co-linearity between SNPs for each trait, we excluded SNPs that were correlated (i.e. *r*^2^ ≥ 0.01) within each trait, and only considered the SNPs with the strongest effect on the trait for inclusion in genetic risk scores (Supplementary Table 2). For type-2 diabetes, linkage disequilibrium (LD) scores with rs140730081 were calculated via a proxy SNP rs2259835 (*r*^2^ = 0.48). After imposing these criteria, we obtained 7 SNPs for 2-h post-challenge glucose, 75 for BMI, 33 for fasting glucose, 13 for fasting insulin, 54 for HDL cholesterol, 26 for LDL cholesterol, 38 for type-2 diabetes, 39 for total cholesterol, 25 for triglycerides and 33 for WHR.

### Glioma association results

To evaluate the association of each genetic instrument with glioma risk, we made use of data from the most recent meta-analysis of GWAS in glioma, comprising >10 million genetic variants (after imputation) in 12,488 glioma patients and 18,169 controls from eight independent GWAS data sets of individuals of European descent (Supplementary Table 3).^20^ Comprehensive details of the genotyping and quality control of the seven GWAS have been previously reported.^20^ To limit the effects of cryptic population stratification, association test statistics for six of the glioma GWAS were generated using principal components as previously detailed.^20^ Gliomas are heterogeneous and different tumour subtypes, defined in part by malignancy grade (e.g. pilocytic astrocytoma World Health Organization (WHO) grade I, diffuse ‘low-grade’ glioma WHO grade II, anaplastic glioma WHO grade III and GBM WHO grade IV) can be distinguished.^26^ For the sake of diagnostic brevity, we considered gliomas as being either GBM or non-GBM tumours.

### Statistical analysis

The odds ratios (OR) of glioma per unit of SD increment for each obesity-related trait, were estimated using generalised summary data-based Mendelian randomisation (GSMR).^27^ This approach performs a multi-SNP MR analysis, which is more powerful than other existing summary data-based MR methodologies.^28^ Separation of signals of causality from horizontal pleiotropy (a single locus influencing affecting multiple phenotypes, also referred to as type-II pleiotropy) is a recognised issue in MR analyses and we therefore used a HEIDI-outlier test^27^ to detect and eliminate genetic instruments that have apparent pleiotropic effects on both the obesity-related trait and glioma. A *P* value threshold of 0.01 for the HEIDI-outlier test was utilised as recommended by Zhu et al. The HEIDI-outlier test may also in theory detect additional violations of the assumptions of MR such as the exclusion restriction assumption. Given that glioma is a binary outcome and type-2 diabetes a binary exposure, the resulting causal effect estimate in this scenario represents the odds for glioma risk per unit increase in the log OR for type-2 diabetes.

For each statistical test, we considered a global significance level of *P* < 0.05 as being satisfactory to derive conclusions. To assess the robustness of our conclusions, we imposed a Bonferroni-corrected significance threshold of 0.0017 (i.e. 0.05/30, to correct for testing 10 traits over three outcomes). We considered a *P* value > 0.05 as non-significant (i.e. no association), a *P* value ≤ 0.05 as evidence for a potential causal association, and a *P* value ≤ 0.0017 as significant evidence for an association. Additionally, we defined the Bayesian false null probability (BFNP) using the Bayesian false discovery probability (BFDP) as per Wakefield^29^ by BFNP = 1 − BFDP. Then to assess whether null results found could be considered reliable, we calculated the minimum prior probability of the alternative hypothesis for which the BFNP was >10%. The power of an MR investigation depends greatly on the proportion of variance in the risk factor that is explained by the respective IV. We estimated study power a priori using the methodology of Burgess.^30^ Statistical analyses were undertaken using R software (Version 3.1.2).

## Results

In our data sets, there were missing data for one fasting insulin SNP (rs1530559), four type-2 diabetes SNPs (rs2972156, rs34706136, rs11257658, rs144613775) and one total cholesterol SNP (rs7570971). These SNPs were excluded from our analysis. Performing HEIDI-outlier analysis on the instruments for each trait identified two SNPs as violating the assumptions of MR with respect to horizontal pleiotropy, rs11603023 for total cholesterol and rs5756931 for triglyceride, which were further excluded. Both SNPs are in LD with the lead SNP in glioma risk loci.

**Fig. 1:**
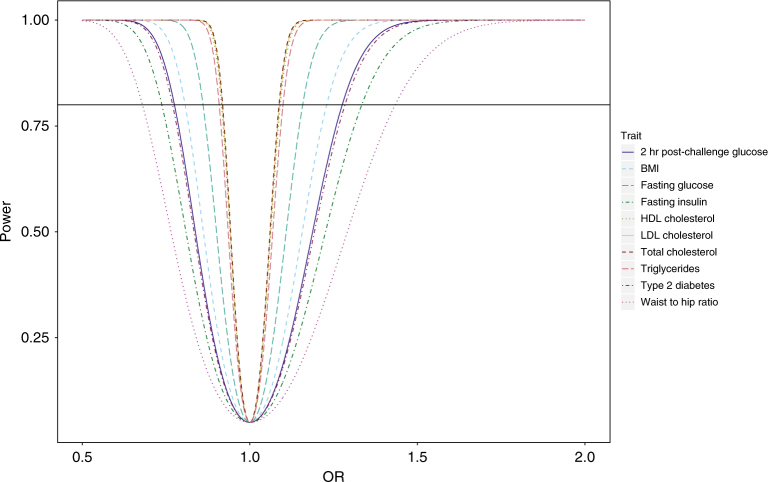
Study power against OR for each obesity-related trait and all glioma (*P* = 0.05, two-sided). A line indicating a power of 80% is shown. BMI body mass index, HDL high-density lipoprotein, LDL low-density lipoprotein, OR odds ratio

Subsequently, Table 1 details the number of SNPs used as an IV for each of the obesity-related traits, the mean and SD of the risk factor in the original discovery study, and the proportion of variance explained for each factor by the corresponding genetic instruments. Effect estimates for each SNP used as genetic instruments for each risk factor and disease risk are detailed in Supplementary Table 1. For BMI and LDL, the SNPs rs12016871 and rs9411489 have since merged with the SNPs rs9581854 and rs635634, respectively, and it is from these subsequent SNPs the associations with glioma were derived. Figure 1 shows the statistical power of genetic instruments for different levels of predicted ORs for each obesity-related trait.

**Table 1:** Metabolic risk factors for which genetic instruments were developed and evaluated in relation to disease risk

Trait	SNPs^a^	Mean (SD)	Units	PVE (%)	References
Two hour post-challenge glucose	7	5.6 (1.7)	mmol/l	1.7	24
BMI	75	27.0 (4.6)	kg/m^2^	2.4	21
Fasting glucose	33	5.2 (0.8)	mmol/l	4.8	24
Fasting insulin	12	56.9 (44.4)	pmol/l	1.2	24
HDL cholesterol	54	53.3 (15.5)	mg/dl	13.7	23
LDL cholesterol	26	133.6 (38.0)	mg/dl	14.6	23
Type-2 diabetes	34	—	—	1.6	25
Total cholesterol	37	213.3 (42.6)	mg/dl	15.0	23
Triglycerides	24	140.9 (87.8)	mg/dl	11.7	23
WHR	33	1.1 (0.1)	cm/cm	0.7	22

*BMI* body mass index, *HDL* high-density lipoprotein, *LDL* low-density lipoprotein, *PVE* proportion of variance explained, *SD* standard deviation, *SNP* single-nucleotide polymorphism, *WHR* waist–hip ratio

^a^Number of SNPs used after quality control

**Fig. 2:**
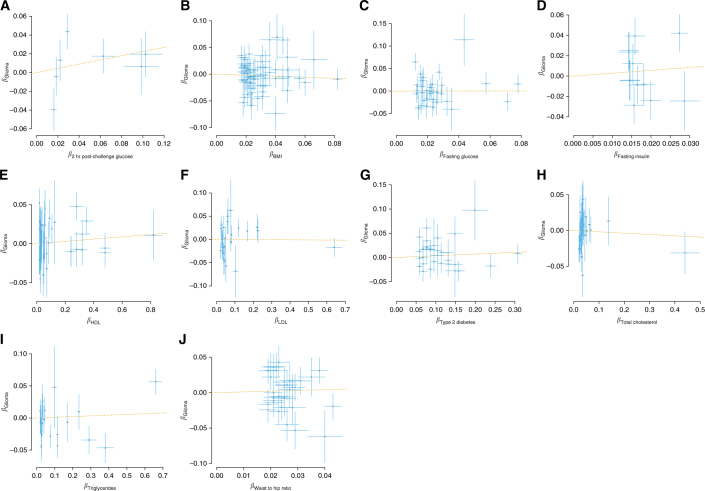
SNP-specific effects for risk of all glioma. For each figure, the effect size of the respective measure for: **a** 2-h post-challenge glucose, **b** BMI, **c** fasting glucose, **d** fasting insulin, **e** HDL cholesterol, **f** LDL cholesterol, **g** type-2 diabetes, **h** total cholesterol, **i** triglycerides and **j** WHR is plotted against the effect for all glioma. Error bars represent one SD. The GSMR estimate is plotted as a dashed line for reference. BMI body mass index, GSMR generalised summary data-based Mendelian randomisation, HDL high-density lipoprotein, LDL low-density lipoprotein, SD standard deviation, WHR waist–hip ratio

Figure 2 shows a plot of the association of each IV with exposure against the association with glioma, together with the resulting GSMR estimate of the log OR. For each of the obesity-related traits under investigation, an approximately null estimate for effect was obtained, with the strongest association being shown by fasting insulin. Setting a threshold of *P* ≤ 0.05, no statistically significant associations were shown for 2-h post-challenge glucose (OR_SD_ = 1.25, 95% confidence interval (CI) = 0.93–1.67), BMI (OR_SD_ = 0.91, 95% CI = 0.77–1.07), fasting glucose (OR_SD_ = 1.00, 95% CI = 0.78–1.3), fasting insulin (OR_SD_ = 1.32, 95% CI = 0.71–2.46), HDL cholesterol (OR_SD_ = 1.01, 95% CI = 0.98–1.05), LDL cholesterol (OR_SD_ = 1.00, 95% CI = 0.95–1.05), type-2 diabetes (OR_SD_ = 1.04, 95% CI = 0.97–1.11), total cholesterol (OR_SD_ = 0.98, 95% CI = 0.88–1.09), triglycerides (OR_SD_ = 1.01, 95% CI = 0.97–1.06) and WHR (OR_SD_ = 1.11, 95% CI = 0.84–1.46).

**Fig. 3:**
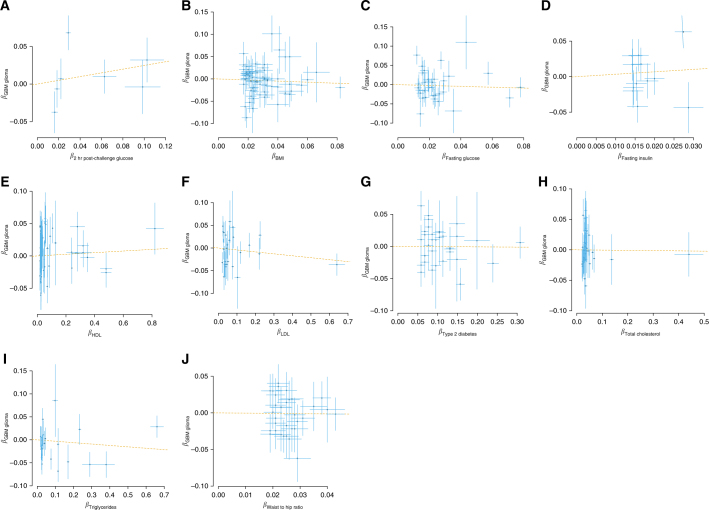
SNP-specific effects for risk of GBM glioma. For each figure, the effect size of the respective measure for **a** 2-h post-challenge glucose, **b** BMI, **c** fasting glucose, **d** fasting insulin, **e** HDL cholesterol, **f** LDL cholesterol, **g** type-2 diabetes, **h** total cholesterol, **i** triglycerides and **j** WHR is plotted against the effect for GBM glioma. Error bars represent one SD. The GSMR estimate is plotted as a dashed line for reference. BMI body mass index, GBM glioblastoma mulitforme, GSMR generalised summary data-based Mendelian randomisation, HDL high-density lipoprotein, LDL low-density lipoprotein, SD standard deviation, WHR waist–hip ratio

**Fig. 4:**
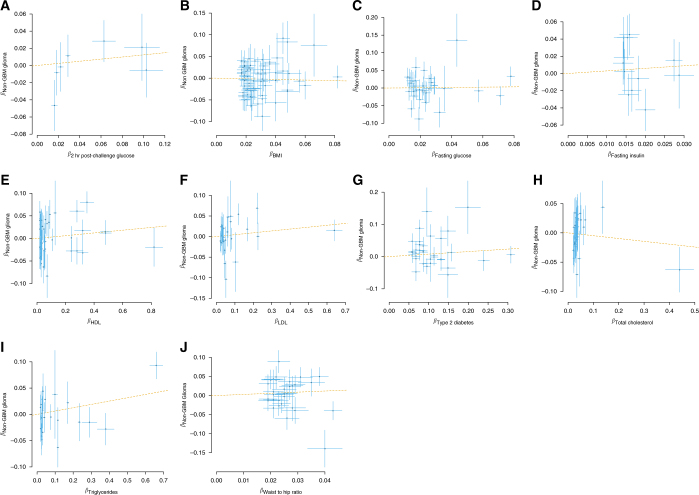
SNP-specific effects for risk of non-GBM glioma. For each figure, the effect size of the respective measure for **a** 2-h post-challenge glucose, **b** BMI, **c** fasting glucose, **d** fasting insulin, **e** HDL cholesterol, **f** LDL cholesterol, **g** type-2 diabetes, **h** total cholesterol, **i** triglycerides and **j** WHR, is plotted against the effect for non-GBM glioma. Error bars represent one SD. The GSMR estimate is plotted as a dashed line for reference. BMI body mass index, GBM glioblastoma mulitforme, GSMR generalised summary data-based Mendelian randomisation, HDL high-density lipoprotein, LDL low-density lipoprotein, SD standard deviation, WHR waist–hip ratio

We explored the possibility that a relationship between an obesity-related trait and glioma might be subtype-specific, considering GBM and non-GBM separately. Figures 3 and 4 show corresponding plots of the association of each IV with exposure against the association with GBM and non-GBM glioma. The strongest association was provided by the relationship between increased triglyceride level and risk of non-GBM glioma (OR_SD_ = 1.07, 95% CI = 1.00–1.13, *P* = 0.044), albeit non-significant after adjustment for multiple testing (Table 2). Table 3 presents the minimum prior probabilities of an association required for each trait to have a BFNP ≥ 0.1. Where possible, the maximum likely OR has been taken from the largest value reported in observational studies.^7, 12, 31^ In the event that this was not possible, an upper bound of 2 was chosen. If the ‘true’ maximum likely OR were lower, then the smallest required prior probability would in fact be lower. There is no current precedent for what value should be taken for the prior probability of an association, indeed attempting to sample published papers would produce an over estimation due to winners curse, but it is noted that a value of 10% would ensure all the results reported would have significance.

**Table 2:** GSMR results for the combined obesity-related IVs

Trait	All glioma		GBM		Non-GBM	
	OR (95% CI)	*P* value	OR (95% CI)	*P* value	OR (95% CI)	*P* value
Two hour post-challenge glucose	1.25 (0.93–1.67)	0.132	1.28 (0.90–1.83)	0.173	1.13 (0.77–1.66)	0.525
BMI	0.91 (0.77–1.07)	0.247	0.89 (0.73–1.08)	0.237	0.93 (0.75–1.15)	0.510
Fasting glucose	1.00 (0.78–1.3)	0.974	0.89 (0.66–1.22)	0.484	1.04 (0.75–1.45)	0.809
Fasting insulin	1.32 (0.71–2.46)	0.374	1.41 (0.66–3.00)	0.377	1.35 (0.60–3.04)	0.471
HDL cholesterol	1.01 (0.98–1.05)	0.375	1.01 (0.97–1.05)	0.532	1.03 (0.99–1.08)	0.167
LDL cholesterol	1.00 (0.95–1.05)	0.939	0.96 (0.90–1.02)	0.197	1.05 (0.98–1.12)	0.195
Type-2 diabetes	1.04 (0.97–1.11)	0.290	1.00 (0.92–1.08)	0.933	1.08 (0.99–1.18)	0.076
Total cholesterol	0.98 (0.88–1.09)	0.736	1.00 (0.87–1.14)	0.949	0.95 (0.83–1.10)	0.505
Triglycerides	1.01 (0.97–1.06)	0.637	0.97 (0.92–1.03)	0.291	1.07 (1.00–1.13)	0.044
WHR	1.11 (0.84–1.46)	0.456	0.97 (0.69–1.35)	0.847	1.34 (0.94–1.93)	0.109

*BMI* body mass index, *CI* confidence interval, *GBM* glioblastoma multiforme, *GSMR* generalised summary data-based Mendelian randomisation, *HDL* high-density lipoprotein, *IV* instrumental variable, *LDL* low-density lipoprotein, *OR* odds ratio, *SD* standard deviation, *WHR* waist–hip ratio

**Table 3:** Prior probability of association required for BFNP > 0.1, for the combined obesity-related IVs

Trait	Glioma		References
	Maximum likely OR	Minimum required prior probability	
Two hour post-challenge glucose	2.00	0.10	N/A
BMI	1.27	0.11	8
Fasting glucose	1.57	0.18	31
Fasting insulin	2.00	0.12	N/A
HDL cholesterol	200	0.64	N/A
LDL cholesterol	2.00	0.61	N/A
Type-2 diabetes	0.60	0.31	12
Total cholesterol	2.00	0.41	N/A
Triglycerides	2.00	0.60	N/A
WHR	2.00	0.19	N/A

*BFNP* Bayesian false null probability, *BMI* body mass index, *HDL* high-density lipoprotein, *IV* instrumental variable, *LDL* low-density lipoprotein, *WHR* waist–hip ratio, *OR* odds ratio, *N/A* no observational data to inform maximum likely OR, value of 2 taken

## Discussion

There is an abundance of studies that have implicated obesity and related traits (notably diabetes), as risk factors for all of the major common cancers, including breast, colorectal, oesophageal, pancreatic, ovarian and renal.^3^ Furthermore, there is increasing evidence that obesity is likely to also be a risk factor for many of the less common tumours, such as those of the haematopoietic system.^3, 32^ The mechanistic basis of how obesity and diabetes affects an increased cancer risk is poorly understood. The long-term metabolic consequences of obesity and its related traits are complex and several mechanisms have been suggested, including increased insulin and insulin-like growth factor signalling, chronic inflammation and signalling via adipokines.^33^ Such mechanisms would be compatible with obesity and related traits having a generic effect on cancer risk.

Evidence for obesity influencing risk of glioma from previous observational studies has been mixed.^4, 6, 9^ Intriguingly, in contrast to other cancers, an inverse relationship between both diabetes and increased HbA1c with risk of glioma has been reported in some but not all studies.^4–7, 9^ Furthermore, in so far as it has been studied, anti-diabetic treatment has been reported to not influence glioma risk.^12^ In terms of the wider spectrum of the metabolic syndrome, a study has linked elevated levels of triglyceride to risk of developing glioma.^9^

Our findings do not support a causal role for higher BMI and related metabolic risk factors, including diagnosis of type-2 diabetes and blood lipid levels, in influencing glioma risk. An important strength of our analysis is that by utilising the random allocation of genetic variants, we were able to overcome potential confounding, for example, from other interrelated traits.^14, 15^ Furthermore, reverse causation and selection bias may have biased estimates from previously published observational studies. By exploiting data from large genetic consortia for multiple obesity-related traits and glioma risk has enabled us to more precisely test study hypotheses than if we had been reliant on individual-level data from a small study. The only obesity-related trait with a first-stage *F*-statistic <10 was WHR (*F* = 6.75) and therefore weak instrument bias for other traits is unlikely.^34^ In addition, given that a poor outcome from glioma is almost universal, it is unlikely that survival bias will have influenced study findings materially. Finally, we have employed a Bayesian approach to interpret the significance of the null results while comparing our findings to published observational epidemiological studies. There is currently no precedent within the MR community as to what value is an accurate representation of the prior probability of association. If the true value is ~20%, then the null findings for 2 h post-challenge glucose, BMI, fasting glucose, fasting insulin and WHR all have a >10% chance of being false.

There are however potential limitations in our analysis that warrant further discussion. Firstly, the use of summary test statistics in two-sample MR analyses requires consideration of sample overlap, the winner’s curse and genotype uncertainty.^35, 36^ Sample overlap between the association studies of the exposure traits and outcome trait has the potential of inflating the type I error rate. The number of controls shared between the glioma GWAS and the anthropometric and lipid GWAS are, however <2% of the respective exposure sample size. Although we are unable to calculate an exact number of glioma cases sampled in the exposure GWAS, given the lifetime risk of glioma is only 0.24%, very few numbers of glioma cases will have been analysed in the exposure trait studies. Hence, such sample overlap is unlikely to contribute to type I error rate inflation.^36^ As the instrumental variables were discovered in the data used in this two-sample MR analysis, weak instrument bias will be accentuated due to winner’s curse, thus attenuating the causal effect estimate towards the null.^36^ Uncertainty with respect to genotyping or disease associations may diminish causal effect estimates.^36^ However IVs used in this analysis are robust and only SNPs passing stringent quality control thresholds were used in the analysis. Secondly, MR is limited in the extent to which it can explore different life course models, such as when an exposure has a temporal relationship to the outcome risk.^35^ Finally, our study does have limitations related to power. However, based on the relatively sizable fraction of variance explained by the genetic instruments for the majority of the obesity-related factors (Table 1), typically there was sufficient statistical power (>80%) to detect even modest odds ratios of 1.43, and close to complete statistical power (99%) to detect relative risks of 1.72 (Fig. 1).

In conclusion, our findings shed light on an issue for which the evidence to date has been mixed. Specifically, they provide evidence against obesity and related traits as significant risk factors for the development of glioma.

### Availability of data and material

Genotype data from the GICC GWAS are available from the database of Genotypes and Phenotypes (dbGaP) under accession phs001319.v1.p1. In addition, genotypes from the GliomaScan GWAS can be accessed through dbGaP accession phs000652.v1.p1.

## Electronic supplementary material

Supplementary Table 1(DOCX 98 kb)

Supplementary Table 2(XLSX 79 kb)

Supplementary Table 3(DOCX 17 kb)

Supplementary Tables Legends(DOCX 13 kb)

## References

[1] Ostrom QT (2013). CBTRUS statistical report: primary brain and central nervous system tumors diagnosed in the United States in 2006–2010. NeuroOncology.

[2] Ostrom QT (2014). The epidemiology of glioma in adults: a “state of the science” review. NeuroOncology.

[3] Kyrgiou M (2017). Adiposity and cancer at major anatomical sites: umbrella review of the literature. BMJ.

[4] Kaplan S, Novikov I, Modan B (1997). Nutritional factors in the etiology of brain tumors: potential role of nitrosamines, fat, and cholesterol. Am. J. Epidemiol..

[5] Niedermaier T (2015). Body mass index, physical activity, and risk of adult meningioma and glioma: a meta-analysis. Neurology.

[6] Sergentanis TN (2015). Obesity and risk for brain/CNS tumors, gliomas and meningiomas: a meta-analysis. PLoS ONE.

[7] Wiedmann M (2013). Body mass index and the risk of meningioma, glioma and schwannoma in a large prospective cohort study (The HUNT Study). Br. J. Cancer.

[8] Dai ZF, Huang QL, Liu HP (2015). Different body mass index grade on the risk of developing glioma: a meta-analysis. Chin. Neurosurg. J..

[9] Edlinger M (2012). Blood pressure and other metabolic syndrome factors and risk of brain tumour in the large population-based Me-Can cohort study. J. Hypertens..

[10] Kitahara CM (2014). Personal history of diabetes, genetic susceptibility to diabetes, and risk of brain glioma: a pooled analysis of observational studies. Cancer Epidemiol. Biomark. Prev..

[11] Schwartzbaum J (2017). Associations between prediagnostic blood glucose levels, diabetes, and glioma. Sci. Rep..

[12] Seliger C (2016). Diabetes, use of anti-diabetic drugs, and the risk of glioma. NeuroOncology.

[13] Zhao L, Zheng Z, Huang P (2016). Diabetes mellitus and the risk of glioma: a meta-analysis. Oncotarget.

[14] Brown CD (2000). Body mass index and the prevalence of hypertension and dyslipidemia. Obes. Res..

[15] GBD 2015 Obesity Collaborators et al. Health effects of overweight and obesity in 195 countries over 25 years. *N. Engl. J. Med.* **377**, 13–27 (2017). 10.1056/NEJMoa1614362 PMC5477817 28604169

[16] Davey Smith G, Hemani G (2014). Mendelian randomization: genetic anchors for causal inference in epidemiological studies. Hum. Mol. Genet..

[17] Nitsch D (2006). Limits to causal inference based on Mendelian randomization: a comparison with randomized controlled trials. Am. J. Epidemiol..

[18] Freeman G, Cowling BJ, Schooling CM (2013). Power and sample size calculations for Mendelian randomization studies using one genetic instrument. Int. J. Epidemiol..

[19] Pierce BL, Burgess S (2013). Efficient design for Mendelian randomization studies: subsample and 2-sample instrumental variable estimators. Am. J. Epidemiol..

[20] Melin BS (2017). Genome-wide association study of glioma subtypes identifies specific differences in genetic susceptibility to glioblastoma and non-glioblastoma tumors. Nat. Genet..

[21] Locke AE (2015). Genetic studies of body mass index yield new insights for obesity biology. Nature.

[22] Shungin D (2015). New genetic loci link adipose and insulin biology to body fat distribution. Nature.

[23] Willer CJ (2013). Discovery and refinement of loci associated with lipid levels. Nat. Genet..

[24] Scott RA (2012). Large-scale association analyses identify new loci influencing glycemic traits and provide insight into the underlying biological pathways. Nat. Genet..

[25] Gaulton KJ (2015). Genetic fine mapping and genomic annotation defines causal mechanisms at type-2 diabetes susceptibility loci. Nat. Genet..

[26] Louis DN (2016). The 2016 World Health Organization Classification of tumors of the central nervous system: a summary. Acta Neuropathol..

[27] Zhu Z. et al. Causal associations between risk factors and common diseases inferred from GWAS summary data. *Nat. Commun.* **9**, 224 (2018). 10.1038/s41467-017-02317-2 PMC5768719 29335400

[28] Burgess S, Butterworth A, Thompson SG (2013). Mendelian randomization analysis with multiple genetic variants using summarized data. Genet. Epidemiol..

[29] Wakefield J (2007). A Bayesian measure of the probability of false discovery in genetic epidemiology studies. Am. J. Hum. Genet..

[30] Burgess S (2014). Sample size and power calculations in Mendelian randomization with a single instrumental variable and a binary outcome. Int. J. Epidemiol..

[31] Derr RL (2009). Association between hyperglycemia and survival in patients with newly diagnosed glioblastoma. J. Clin. Oncol..

[32] Yang TO (2016). Body size in early life and risk of lymphoid malignancies and histological subtypes in adulthood. Int J. Cancer.

[33] Font-Burgada J, Sun B, Karin M (2016). Obesity and cancer: the oil that feeds the flame. Cell Metab..

[34] Lawlor DA, Harbord RM, Sterne JA, Timpson N, Davey Smith G (2008). Mendelian randomization: using genes as instruments for making causal inferences in epidemiology. Stat. Med..

[35] Lawlor DA (2016). Commentary: two-sample Mendelian randomization: opportunities and challenges. Int. J. Epidemiol..

[36] Burgess S, Davies NM, Thompson SG (2016). Bias due to participant overlap in two-sample Mendelian randomization. Genet. Epidemiol..

