# Supplementary material for: Influence of obesity-related risk factors in the aetiology of glioma
Source: Br J Cancer. 2018 Mar 13;118(7):1020–7. doi: 10.1038/s41416-018-0009-x (PMC5931112; doi:10.1038/s41416-018-0009-x)
Supplement: Supplementary file 1 — Supplementary Table 1(DOCX 98 kb) [file 41416_2018_9_MOESM1_ESM.docx]

**Supplementary Table 1: Effect allele, frequency, effect on obesity-related trait and strength of association with glioma for SNPs used as genetic instruments.**

2-hour post-challenge glucose

| **Chromosome** | **SNP** | **Base pair position*** | **Alleles**** | **MAF** | **2-hour post-challenge glucose** | **All glioma** | **GBM** | **Non-GBM** |
| --- | --- | --- | --- | --- | --- | --- | --- | --- |
|  |  |  |  |  | **OR (95% CI)** | **OR (95% CI)** | **OR (95% CI)** | **OR (95% CI)** |
| 2 | rs1260326 | 27730940 | T/C | T=0.378 | 1.03 (1.03-1.03) | 1.04 (1.01-1.08) | 1.07 (1.02-1.12) | 1.01 (0.96-1.06) |
| 3 | rs11717195 | 123082398 | C/T | C=0.210 | 1.02 (1.02-1.03) | 1.01 (0.97-1.06) | 1.01 (0.96-1.06) | 1 (0.94-1.06) |
| 5 | rs1019503 | 96254817 | G/A | A=0.483 | 1.06 (1.04-1.09) | 1.02 (0.98-1.05) | 1.01 (0.97-1.06) | 1.03 (0.98-1.08) |
| 7 | rs6975024 | 44231886 | T/C | C=0.150 | 1.11 (1.07-1.14) | 1.02 (0.97-1.07) | 1.03 (0.97-1.09) | 0.99 (0.93-1.06) |
| 8 | rs11782386 | 9201787 | T/C | T=0.103 | 1.10 (1.07-1.14) | 1.01 (0.95-1.07) | 1.00 (0.93-1.07) | 1.02 (0.95-1.10) |
| 10 | rs12255372 | 114808902 | G/T | T=0.236 | 1.02 (1.01-1.02) | 1.00 (0.96-1.04) | 0.99 (0.95-1.04) | 0.99 (0.94-1.04) |
| 19 | rs11672660 | 46180184 | T/C | T=0.220 | 1.02 (1.01-1.02) | 0.96 (0.92-1.00) | 0.96 (0.91-1.02) | 0.95 (0.90-1.01) |

BMI

| **Chromosome** | **SNP** | **Base pair position *** | **Alleles**** | **MAF** | **BMI** | **All glioma** | **GBM** | **Non-GBM** |
| --- | --- | --- | --- | --- | --- | --- | --- | --- |
|  |  |  |  |  | **OR (95% CI)** | **OR (95% CI)** | **OR (95% CI)** | **OR (95% CI)** |
| 1 | rs543874 | 177889480 | A/G | G=0.193 | 1.05 (1.04-1.06) | 0.97 (0.93-1.01) | 0.97 (0.91-1.02) | 0.97 (0.92-1.03) |
| 1 | rs3101336 | 72751185 | T/C | T=0.387 | 1.03 (1.03-1.04) | 0.99 (0.96-1.03) | 1.00 (0.95-1.04) | 1.01 (0.96-1.06) |
| 1 | rs12566985 | 75002193 | A/G | G=0.446 | 1.02 (1.02-1.03) | 0.97 (0.94-1.01) | 0.98 (0.94-1.02) | 0.97 (0.93-1.02) |
| 1 | rs17024393 | 110154688 | T/C | C=0.040 | 1.07 (1.05-1.09) | 1.03 (0.92-1.14) | 1.01 (0.89-1.16) | 1.08 (0.94-1.24) |
| 1 | rs11165643 | 96924097 | C/T | C=0.417 | 1.02 (1.02-1.03) | 1.02 (0.98-1.06) | 1.02 (0.97-1.06) | 1.02 (0.98-1.07) |
| 1 | rs12401738 | 78446761 | G/A | A=0.352 | 1.02 (1.01-1.03) | 1.01 (0.97-1.05) | 1.02 (0.97-1.06) | 1.02 (0.97-1.07) |
| 1 | rs657452 | 49589847 | G/A | A=0.394 | 1.02 (1.02-1.03) | 0.98 (0.95-1.02) | 1.00 (0.96-1.05) | 0.96 (0.91-1.01) |
| 1 | rs2820292 | 201784287 | A/C | A=0.445 | 1.02 (1.01-1.03) | 1.01 (0.97-1.04) | 1.01 (0.97-1.05) | 1.02 (0.97-1.06) |
| 2 | rs13021737 | 632348 | A/G | A=0.172 | 1.06 (1.05-1.07) | 0.98 (0.94-1.03) | 1.00 (0.94-1.06) | 0.98 (0.92-1.05) |
| 2 | rs10182181 | 25150296 | A/G | G=0.462 | 1.03 (1.03-1.04) | 0.97 (0.93-1.00) | 0.97 (0.93-1.01) | 0.95 (0.91-1.00) |
| 2 | rs1016287 | 59305625 | C/T | T=0.287 | 1.02 (1.02-1.03) | 0.94 (0.90-0.99) | 0.96 (0.9-1.02) | 0.94 (0.88-1.00) |
| 2 | rs2121279 | 143043285 | C/T | T=0.152 | 1.03 (1.02-1.03) | 1.00 (0.94-1.05) | 1.02 (0.95-1.09) | 0.98 (0.91-1.05) |
| 2 | rs7599312 | 213413231 | A/G | A=0.276 | 1.02 (1.02-1.03) | 0.99 (0.95-1.03) | 0.99 (0.95-1.04) | 0.98 (0.93-1.04) |
| 2 | rs11126666 | 26928811 | G/A | A=0.283 | 1.02 (1.01-1.03) | 1.00 (0.96-1.04) | 1.01 (0.96-1.06) | 1.00 (0.95-1.06) |
| 2 | rs1528435 | 181550962 | C/T | C=0.369 | 1.02 (1.01-1.02) | 1.01 (0.97-1.05) | 1.00 (0.96-1.05) | 1.02 (0.98-1.08) |
| 2 | rs11688816 | 63053048 | A/G | A=0.475 | 1.02 (1.01-1.02) | 1.00 (0.97-1.04) | 0.99 (0.95-1.03) | 1.02 (0.97-1.07) |
| 3 | rs1516725 | 185824004 | T/C | T=0.128 | 1.05 (1.04-1.06) | 1.07 (1.01-1.12) | 1.05 (0.98-1.12) | 1.10 (1.02-1.18) |
| 3 | rs13078960 | 85807590 | T/G | G=0.196 | 1.03 (1.02-1.04) | 1.01 (0.97-1.06) | 1.03 (0.97-1.08) | 1.01 (0.95-1.07) |
| 3 | rs2365389 | 61236462 | T/C | T=0.418 | 1.02 (1.01-1.03) | 1.00 (0.97-1.04) | 1.02 (0.98-1.07) | 0.97 (0.93-1.02) |
| 3 | rs16851483 | 141275436 | G/T | T=0.066 | 1.05 (1.03-1.07) | 1.01 (0.94-1.08) | 1.05 (0.96-1.15) | 0.97 (0.88-1.07) |
| 3 | rs6804842 | 25106437 | A/G | A=0.425 | 1.02 (1.01-1.03) | 1.03 (0.99-1.07) | 1.03 (0.98-1.07) | 1.03 (0.98-1.08) |
| 3 | rs3849570 | 81792112 | C/A | A=0.359 | 1.02 (1.01-1.03) | 1.01 (0.98-1.05) | 1.01 (0.97-1.06) | 1.00 (0.96-1.05) |
| 4 | rs10938397 | 45182527 | A/G | G=0.434 | 1.04 (1.03-1.05) | 0.99 (0.96-1.03) | 0.98 (0.94-1.03) | 0.99 (0.95-1.04) |
| 4 | rs13107325 | 103188709 | C/T | T=0.072 | 1.05 (1.04-1.06) | 1.03 (0.96-1.11) | 0.99 (0.91-1.08) | 1.09 (0.99-1.19) |
| 4 | rs17001654 | 77129568 | C/G | G=0.153 | 1.03 (1.02-1.04) | 0.96 (0.91-1.01) | 1.00 (0.94-1.06) | 0.92 (0.86-0.98) |
| 4 | rs11727676 | 145659064 | C/T | C=0.090 | 1.04 (1.02-1.05) | 1.07 (1.00-1.14) | 1.11 (1.02-1.20) | 1.03 (0.95-1.12) |
| 5 | rs2112347 | 75015242 | G/T | G=0.371 | 1.03 (1.02-1.03) | 0.99 (0.95-1.03) | 1.00 (0.95-1.04) | 0.98 (0.93-1.02) |
| 6 | rs2207139 | 50845490 | A/G | G=0.177 | 1.05 (1.04-1.05) | 0.99 (0.95-1.04) | 0.97 (0.91-1.03) | 1.02 (0.96-1.08) |
| 6 | rs205262 | 34563164 | A/G | G=0.273 | 1.02 (1.02-1.03) | 0.98 (0.94-1.02) | 0.96 (0.92-1.01) | 0.99 (0.94-1.04) |
| 6 | rs13191362 | 163033350 | G/A | G=0.121 | 1.03 (1.02-1.04) | 1.00 (0.94-1.05) | 0.99 (0.92-1.06) | 1.01 (0.94-1.09) |
| 6 | rs2033529 | 40348653 | A/G | G=0.293 | 1.02 (1.01-1.03) | 1.02 (0.98-1.06) | 1.02 (0.97-1.07) | 1.03 (0.98-1.08) |
| 6 | rs9400239 | 108977663 | C/T | T=0.312 | 1.02 (1.01-1.03) | 0.99 (0.95-1.03) | 1.01 (0.96-1.06) | 0.95 (0.90-1.00) |
| 7 | rs1167827 | 75163169 | G/A | A=0.447 | 1.02 (1.01-1.03) | 0.98 (0.94-1.01) | 0.96 (0.92-1.01) | 0.99 (0.95-1.04) |
| 7 | rs2245368 | 76608143 | T/C | C=0.180 | 1.03 (1.02-1.04) | 1.01 (0.96-1.06) | 1.00 (0.94-1.06) | 1.02 (0.96-1.09) |
| 8 | rs17405819 | 76806584 | C/T | C=0.300 | 1.02 (1.02-1.03) | 1.01 (0.97-1.05) | 1.00 (0.95-1.05) | 1.01 (0.96-1.06) |
| 8 | rs2033732 | 85079709 | C/T | T=0.253 | 1.02 (1.01-1.03) | 1.01 (0.97-1.05) | 1.03 (0.98-1.09) | 0.97 (0.92-1.03) |
| 9 | rs10968576 | 28414339 | G/A | G=0.320 | 1.03 (1.02-1.03) | 1.03 (0.99-1.07) | 1.01 (0.96-1.06) | 1.04 (0.99-1.09) |
| 9 | rs1928295 | 120378483 | C/T | C=0.452 | 1.02 (1.01-1.03) | 1.00 (0.97-1.04) | 1.01 (0.97-1.06) | 1.00 (0.95-1.04) |
| 9 | rs4740619 | 15634326 | C/T | C=0.458 | 1.02 (1.01-1.02) | 1.02 (0.98-1.06) | 1.02 (0.98-1.07) | 1.01 (0.97-1.06) |
| 9 | rs10733682 | 129460914 | G/A | A=0.478 | 1.02 (1.01-1.02) | 1.04 (1.00-1.07) | 1.03 (0.99-1.08) | 1.04 (0.99-1.09) |
| 9 | rs6477694 | 111932342 | T/C | C=0.365 | 1.02 (1.01-1.02) | 1.00 (0.97-1.04) | 0.98 (0.94-1.03) | 1.03 (0.98-1.08) |
| 10 | rs7903146 | 114758349 | T/C | T=0.287 | 1.02 (1.02-1.03) | 0.99 (0.95-1.03) | 0.99 (0.95-1.04) | 0.99 (0.94-1.05) |
| 10 | rs17094222 | 102395440 | C/T | C=0.211 | 1.03 (1.02-1.03) | 1.02 (0.98-1.07) | 1.03 (0.98-1.09) | 1.02 (0.96-1.08) |
| 10 | rs11191560 | 104869038 | C/T | C=0.089 | 1.03 (1.02-1.04) | 0.93 (0.87-0.99) | 0.94 (0.87-1.02) | 0.94 (0.87-1.03) |
| 10 | rs7899106 | 87410904 | G/A | G=0.052 | 1.04 (1.03-1.06) | 1.07 (0.99-1.17) | 1.07 (0.96-1.18) | 1.05 (0.94-1.17) |
| 11 | rs11030104 | 27684517 | G/A | G=0.208 | 1.04 (1.03-1.05) | 1.01 (0.97-1.05) | 0.99 (0.94-1.05) | 1.04 (0.98-1.10) |
| 11 | rs3817334 | 47650993 | T/C | T=0.407 | 1.03 (1.02-1.03) | 0.96 (0.93-1.00) | 0.95 (0.91-0.99) | 0.96 (0.92-1.01) |
| 11 | rs4256980 | 8673939 | G/C | C=0.354 | 1.02 (1.01-1.03) | 1.04 (1.00-1.08) | 1.03 (0.98-1.08) | 1.05 (1.00-1.10) |
| 11 | rs12286929 | 115022404 | G/A | A=0.477 | 1.02 (1.02-1.03) | 1.02 (0.98-1.05) | 1.02 (0.98-1.07) | 1.00 (0.95-1.04) |
| 11 | rs2176598 | 43864278 | C/T | T=0.251 | 1.02 (1.01-1.03) | 1.00 (0.96-1.04) | 0.99 (0.94-1.04) | 1.03 (0.97-1.09) |
| 12 | rs7138803 | 50247468 | A/G | A=0.384 | 1.03 (1.03-1.04) | 1.04 (1.00-1.07) | 1.02 (0.98-1.07) | 1.04 (0.99-1.09) |
| 12 | rs11057405 | 122781897 | A/G | A=0.099 | 1.03 (1.02-1.04) | 1.01 (0.95-1.08) | 1.00 (0.92-1.07) | 0.98 (0.91-1.07) |
| 13 | rs12429545 | 54102206 | A/G | A=0.133 | 1.03 (1.02-1.04) | 1.00 (0.95-1.05) | 1.00 (0.94-1.07) | 0.99 (0.93-1.06) |
| 13 | rs12016871 | 28017782 | T/C | T=0.203 | 1.03 (1.02-1.04) | 0.97 (0.92-1.01) | 0.97 (0.92-1.03) | 0.96 (0.90-1.02) |
| 14 | rs7141420 | 79899454 | T/C | C=0.473 | 1.02 (1.02-1.03) | 0.99 (0.96-1.03) | 0.97 (0.93-1.02) | 1.01 (0.96-1.06) |
| 14 | rs11847697 | 30515112 | T/C | T=0.042 | 1.05 (1.03-1.07) | 0.98 (0.90-1.07) | 0.94 (0.84-1.04) | 1.02 (0.91-1.14) |
| 14 | rs10132280 | 25928179 | A/C | A=0.318 | 1.02 (1.02-1.03) | 0.99 (0.95-1.03) | 1.02 (0.97-1.07) | 0.98 (0.93-1.03) |
| 14 | rs12885454 | 29736838 | A/C | A=0.358 | 1.02 (1.01-1.03) | 0.98 (0.94-1.02) | 0.96 (0.92-1.01) | 1.00 (0.95-1.05) |
| 15 | rs16951275 | 68077168 | C/T | C=0.216 | 1.03 (1.02-1.04) | 0.99 (0.95-1.03) | 1.00 (0.95-1.05) | 0.96 (0.91-1.01) |
| 15 | rs3736485 | 51748610 | G/A | A=0.454 | 1.02 (1.01-1.02) | 0.99 (0.95-1.03) | 0.98 (0.94-1.03) | 1.00 (0.95-1.05) |
| 16 | rs1558902 | 53803574 | A/T | A=0.415 | 1.09 (1.08-1.09) | 0.98 (0.94-1.02) | 1.00 (0.96-1.05) | 0.96 (0.92-1.01) |
| 16 | rs3888190 | 28889486 | A/C | A=0.403 | 1.03 (1.03-1.04) | 1.01 (0.97-1.05) | 1.00 (0.96-1.05) | 1.01 (0.96-1.06) |
| 16 | rs12446632 | 19935389 | A/G | A=0.135 | 1.04 (1.03-1.05) | 0.99 (0.95-1.05) | 1.01 (0.95-1.08) | 0.97 (0.91-1.04) |
| 16 | rs758747 | 3627358 | T/C | T=0.265 | 1.02 (1.02-1.03) | 1.00 (0.96-1.04) | 1.01 (0.96-1.06) | 0.98 (0.93-1.04) |
| 16 | rs9925964 | 31129895 | G/A | G=0.380 | 1.02 (1.01-1.03) | 0.99 (0.95-1.03) | 1.01 (0.96-1.05) | 0.97 (0.92-1.02) |
| 17 | rs12940622 | 78615571 | A/G | A=0.425 | 1.02 (1.01-1.02) | 0.96 (0.92-0.99) | 0.92 (0.88-0.96) | 0.99 (0.95-1.04) |
| 17 | rs1000940 | 5283252 | G/A | G=0.32 | 1.02 (1.01-1.03) | 0.99 (0.95-1.03) | 0.99 (0.94-1.03) | 0.99 (0.94-1.05) |
| 18 | rs6567160 | 57829135 | C/T | C=0.236 | 1.06 (1.05-1.07) | 1.03 (0.99-1.08) | 1.06 (1.01-1.11) | 1.01 (0.96-1.07) |
| 18 | rs1808579 | 21104888 | T/C | T=0.466 | 1.02 (1.01-1.02) | 0.97 (0.94-1.01) | 0.97 (0.93-1.02) | 0.98 (0.93-1.02) |
| 18 | rs7243357 | 56883319 | G/T | G=0.188 | 1.02 (1.01-1.03) | 1.00 (0.96-1.05) | 0.98 (0.93-1.04) | 1.04 (0.98-1.10) |
| 19 | rs2287019 | 46202172 | T/C | T=0.196 | 1.04 (1.03-1.05) | 0.96 (0.91-1.00) | 0.96 (0.91-1.02) | 0.94 (0.89-1.00) |
| 19 | rs3810291 | 47569003 | A/G | G=0.334 | 1.03 (1.02-1.04) | 1.00 (0.96-1.03) | 1.02 (0.97-1.06) | 0.98 (0.93-1.03) |
| 19 | rs2075650 | 45395619 | G/A | G=0.152 | 1.03 (1.02-1.04) | 0.95 (0.90-1.00) | 0.94 (0.88-1.00) | 0.96 (0.90-1.03) |
| 19 | rs29941 | 34309532 | G/A | A=0.331 | 1.02 (1.01-1.02) | 0.98 (0.95-1.02) | 1.01 (0.97-1.06) | 0.95 (0.91-1.00) |

Fasting glucose

| **Chromosome** | **SNP** | **Base pair position *** | **Alleles**** | **MAF** | **Fasting glucose** | **All glioma** | **GBM** | **Non-GBM** |
| --- | --- | --- | --- | --- | --- | --- | --- | --- |
|  |  |  |  |  | **OR (95% CI)** | **OR (95% CI)** | **OR (95% CI)** | **OR (95% CI)** |
| 1 | rs340874 | 214159256 | T/C | T=0.479 | 1.01 (1.01-1.02) | 1.00 (0.97-1.04) | 0.99 (0.94-1.03) | 1.02 (0.97-1.07) |
| 2 | rs780094 | 27741237 | T/C | T=0.390 | 1.03 (1.02-1.03) | 1.04 (1.01-1.08) | 1.07 (1.02-1.11) | 1.02 (0.97-1.07) |
| 2 | rs560887 | 169763148 | T/C | T=0.296 | 1.07 (1.07-1.08) | 0.98 (0.94-1.02) | 0.97 (0.92-1.01) | 0.98 (0.93-1.03) |
| 3 | rs7651090 | 185513392 | A/G | G=0.306 | 1.01 (1.01-1.02) | 0.99 (0.96-1.03) | 0.99 (0.95-1.04) | 0.99 (0.94-1.04) |
| 3 | rs11715915 | 49455330 | T/C | T=0.325 | 1.01 (1.01-1.02) | 1.07 (1.03-1.11) | 1.08 (1.03-1.13) | 1.03 (0.98-1.08) |
| 3 | rs11708067 | 123065778 | G/A | G=0.210 | 1.02 (1.02-1.03) | 1.02 (0.97-1.06) | 1.01 (0.96-1.07) | 0.99 (0.94-1.05) |
| 3 | rs1280 | 170713290 | C/T | C=0.136 | 1.03 (1.02-1.03) | 1.00 (0.95-1.05) | 0.98 (0.92-1.04) | 1.03 (0.96-1.10) |
| 5 | rs4869272 | 95539448 | C/T | C=0.311 | 1.02 (1.01-1.02) | 0.98 (0.94-1.02) | 0.97 (0.92-1.01) | 0.99 (0.95-1.05) |
| 6 | rs9368222 | 20686996 | C/A | A=0.283 | 1.01 (1.01-1.02) | 0.96 (0.91-1.02) | 0.93 (0.87-0.99) | 1.01 (0.94-1.09) |
| 7 | rs6943153 | 50791579 | C/T | T=0.335 | 1.02 (1.01-1.02) | 1.04 (1.00-1.08) | 1.05 (1.00-1.10) | 1.02 (0.97-1.07) |
| 7 | rs2191349 | 15064309 | G/T | G=0.474 | 1.03 (1.03-1.03) | 1.01 (0.98-1.05) | 1.01 (0.97-1.06) | 1.01 (0.96-1.06) |
| 7 | rs2908289 | 44223942 | G/A | A=0.163 | 1.06 (1.05-1.07) | 1.02 (0.97-1.07) | 1.03 (0.97-1.09) | 0.99 (0.93-1.06) |
| 8 | rs983309 | 9177732 | G/T | T=0.117 | 1.03 (1.02-1.03) | 0.98 (0.92-1.03) | 0.96 (0.89-1.03) | 1.00 (0.93-1.08) |
| 8 | rs11558471 | 118185733 | G/A | G=0.319 | 1.03 (1.02-1.03) | 0.99 (0.96-1.03) | 0.98 (0.94-1.03) | 1.01 (0.96-1.06) |
| 9 | rs10811661 | 22134094 | C/T | C=0.180 | 1.02 (1.02-1.03) | 0.98 (0.94-1.03) | 0.97 (0.92-1.03) | 0.99 (0.93-1.05) |
| 9 | rs16913693 | 111680359 | G/T | G=0.027 | 1.04 (1.03-1.06) | 1.12 (1.00-1.25) | 1.12 (0.97-1.28) | 1.15 (0.99-1.33) |
| 9 | rs3829109 | 139256766 | A/G | A=0.293 | 1.02 (1.01-1.02) | 1.03 (0.99-1.08) | 1.01 (0.95-1.06) | 1.06 (1.00-1.12) |
| 9 | rs10814916 | 4293150 | A/C | A=0.492 | 1.02 (1.01-1.02) | 0.97 (0.93-1.00) | 0.97 (0.92-1.01) | 0.99 (0.94-1.04) |
| 10 | rs11195502 | 113039667 | T/C | T=0.087 | 1.03 (1.03-1.04) | 0.98 (0.92-1.04) | 1.02 (0.95-1.10) | 0.93 (0.86-1.01) |
| 10 | rs7903146 | 114758349 | C/T | T=0.280 | 1.02 (1.02-1.03) | 1.01 (0.97-1.05) | 1.01 (0.96-1.06) | 1.01 (0.96-1.06) |
| 11 | rs11603334 | 72432985 | A/G | A=0.167 | 1.02 (1.01-1.03) | 0.97 (0.92-1.02) | 1.01 (0.95-1.08) | 0.92 (0.86-0.98) |
| 11 | rs11607883 | 45839709 | A/G | G=0.482 | 1.02 (1.02-1.03) | 1.00 (0.96-1.03) | 0.99 (0.95-1.04) | 0.99 (0.95-1.04) |
| 11 | rs11039182 | 47346723 | C/T | C=0.270 | 1.02 (1.02-1.03) | 0.96 (0.93-1.00) | 0.96 (0.92-1.01) | 0.96 (0.91-1.01) |
| 11 | rs174576 | 61603510 | A/C | A=0.348 | 1.02 (1.02-1.02) | 0.98 (0.95-1.02) | 0.99 (0.95-1.04) | 0.97 (0.92-1.02) |
| 11 | rs10830963 | 92708710 | C/G | G=0.290 | 1.08 (1.08-1.09) | 1.02 (0.97-1.06) | 0.99 (0.94-1.04) | 1.03 (0.98-1.09) |
| 12 | rs10747083 | 133041618 | G/A | G=0.337 | 1.01 (1.01-1.02) | 1.01 (0.98-1.05) | 1.00 (0.96-1.05) | 1.03 (0.98-1.08) |
| 13 | rs11619319 | 28487599 | A/G | G=0.226 | 1.02 (1.01-1.02) | 1.00 (0.96-1.05) | 1.01 (0.96-1.06) | 1.00 (0.94-1.05) |
| 13 | rs576674 | 33554302 | A/G | G=0.154 | 1.02 (1.01-1.02) | 1.00 (0.95-1.05) | 0.98 (0.93-1.04) | 1.01 (0.94-1.07) |
| 14 | rs3783347 | 100839261 | T/G | T=0.211 | 1.02 (1.01-1.02) | 1.02 (0.98-1.07) | 1.04 (0.98-1.09) | 1.02 (0.97-1.08) |
| 15 | rs4502156 | 62383155 | C/T | C=0.448 | 1.02 (1.02-1.03) | 1.00 (0.96-1.04) | 0.97 (0.93-1.02) | 1.05 (1.00-1.10) |
| 19 | rs2302593 | 46196634 | G/C | G=0.497 | 1.01 (1.01-1.02) | 0.96 (0.93-1.00) | 0.98 (0.94-1.02) | 0.94 (0.90-0.99) |
| 20 | rs6113722 | 22557099 | A/G | A=0.043 | 1.04 (1.03-1.05) | 0.96 (0.87-1.05) | 0.93 (0.84-1.04) | 1.00 (0.88-1.13) |
| 20 | rs6072275 | 39743905 | G/A | A=0.165 | 1.02 (1.01-1.02) | 1.01 (0.96-1.06) | 1.03 (0.97-1.10) | 0.98 (0.92-1.04) |

Fasting insulin

| **Chromosome** | **SNP** | **Base pair position *** | **Alleles**** | **MAF** | **Fasting insulin** | **All glioma** | **GBM** | **Non-GBM** |
| --- | --- | --- | --- | --- | --- | --- | --- | --- |
|  |  |  |  |  | **OR (95% CI)** | **OR (95% CI)** | **OR (95% CI)** | **OR (95% CI)** |
| 1 | rs2820436 | 219640680 | A/C | A=0.329 | 1.02 (1.01-1.02) | 1.01 (0.97-1.05) | 0.99 (0.94-1.04) | 1.04 (0.99-1.10) |
| 2 | rs10195252 | 165513091 | C/T | C=0.407 | 1.02 (1.01-1.02) | 1.04 (1.00-1.08) | 1.03 (0.99-1.08) | 1.05 (1.00-1.10) |
| 2 | rs2972143 | 227116365 | A/G | A=0.376 | 1.01 (1.01-1.02) | 1.02 (0.99-1.06) | 1.03 (0.98-1.08) | 1.01 (0.96-1.06) |
| 2 | rs780094 | 27741237 | T/C | T=0.393 | 1.03 (1.02-1.03) | 1.04 (1.01-1.08) | 1.07 (1.02-1.11) | 1.02 (0.97-1.07) |
| 4 | rs9884482 | 106081636 | T/C | C=0.390 | 1.02 (1.01-1.02) | 0.99 (0.96-1.03) | 1.02 (0.97-1.06) | 0.98 (0.93-1.03) |
| 5 | rs4865796 | 53272664 | G/A | G=0.327 | 1.01 (1.01-1.02) | 1.00 (0.96-1.03) | 0.98 (0.94-1.03) | 1.00 (0.95-1.05) |
| 6 | rs2745353 | 127452935 | C/T | C=0.492 | 1.01 (1.01-1.02) | 1.03 (0.99-1.06) | 1.02 (0.97-1.06) | 1.04 (0.99-1.09) |
| 7 | rs1167800 | 75176196 | G/A | G=0.457 | 1.02 (1.01-1.02) | 0.97 (0.94-1.01) | 0.96 (0.92-1.00) | 0.98 (0.93-1.02) |
| 8 | rs983309 | 9177732 | G/T | T=0.116 | 1.03 (1.02-1.04) | 0.98 (0.92-1.03) | 0.96 (0.89-1.03) | 1.00 (0.93-1.08) |
| 10 | rs7903146 | 114758349 | T/C | T=0.281 | 1.02 (1.01-1.02) | 0.99 (0.95-1.03) | 0.99 (0.95-1.04) | 0.99 (0.94-1.05) |
| 16 | rs1421085 | 53800954 | T/C | C=0.417 | 1.02 (1.02-1.03) | 0.98 (0.94-1.01) | 1.00 (0.95-1.04) | 0.96 (0.91-1.01) |
| 19 | rs731839 | 33899065 | A/G | G=0.337 | 1.01 (1.01-1.02) | 1.00 (0.96-1.03) | 0.98 (0.94-1.03) | 1.02 (0.97-1.07) |

HDL cholesterol

| **Chromosome** | **SNP** | **Base pair position *** | **Alleles**** | **MAF** | **HDL** | **All glioma** | **GBM** | **Non-GBM** |
| --- | --- | --- | --- | --- | --- | --- | --- | --- |
|  |  |  |  |  | **OR (95% CI)** | **OR (95% CI)** | **OR (95% CI)** | **OR (95% CI)** |
| 1 | rs12748152 | 27138393 | T/C | T=0.090 | 1.05 (1.04-1.07) | 0.98 (0.92-1.05) | 0.98 (0.91-1.07) | 0.97 (0.89-1.06) |
| 1 | rs12145743 | 156700651 | T/G | G=0.340 | 1.02 (1.01-1.03) | 1.03 (0.99-1.07) | 1.05 (1.00-1.10) | 1.02 (0.97-1.07) |
| 1 | rs4650994 | 178515312 | A/G | G=0.490 | 1.02 (1.01-1.03) | 1.03 (0.99-1.06) | 1.01 (0.97-1.06) | 1.04 (1.00-1.09) |
| 1 | rs4660293 | 40028180 | G/A | G=0.240 | 1.04 (1.03-1.04) | 1.00 (0.96-1.04) | 1.01 (0.96-1.06) | 0.99 (0.94-1.05) |
| 1 | rs1689800 | 182168885 | G/A | G=0.350 | 1.03 (1.03-1.04) | 1.00 (0.96-1.04) | 1.02 (0.98-1.07) | 0.98 (0.93-1.03) |
| 1 | rs4846914 | 230295691 | G/A | G=0.410 | 1.62 (1.51-1.73) | 0.99 (0.95-1.03) | 0.97 (0.93-1.02) | 1.02 (0.97-1.07) |
| 2 | rs12328675 | 165540800 | T/C | C=0.130 | 1.05 (1.03-1.06) | 0.96 (0.91-1.01) | 0.98 (0.92-1.04) | 0.94 (0.88-1.01) |
| 2 | rs2972146 | 227100698 | G/T | G=0.370 | 1.03 (1.02-1.04) | 1.02 (0.99-1.06) | 1.03 (0.99-1.08) | 1.01 (0.96-1.06) |
| 3 | rs2606736 | 11400249 | T/C | C=0.390 | 1.03 (1.02-1.03) | 1.03 (0.99-1.07) | 1.04 (0.99-1.09) | 1.02 (0.98-1.08) |
| 3 | rs2290547 | 47061183 | A/G | A=0.200 | 1.03 (1.02-1.04) | 1.02 (0.97-1.07) | 1.02 (0.96-1.08) | 1.02 (0.96-1.08) |
| 3 | rs2013208 | 50129399 | C/T | T=0.500 | 1.03 (1.02-1.03) | 0.96 (0.92-0.99) | 0.94 (0.90-0.98) | 0.98 (0.93-1.02) |
| 3 | rs13326165 | 52532118 | G/A | A=0.210 | 1.03 (1.02-1.04) | 1.00 (0.96-1.04) | 1.04 (0.99-1.10) | 0.95 (0.89-1.00) |
| 4 | rs10019888 | 26062990 | G/A | G=0.180 | 1.03 (1.02-1.04) | 0.99 (0.94-1.04) | 0.96 (0.91-1.02) | 1.03 (0.97-1.09) |
| 4 | rs3822072 | 89741269 | A/G | A=0.460 | 1.03 (1.02-1.03) | 1.00 (0.96-1.04) | 0.98 (0.94-1.03) | 1.02 (0.97-1.07) |
| 4 | rs2602836 | 100014805 | G/A | A=0.440 | 1.02 (1.01-1.03) | 0.98 (0.94-1.02) | 0.97 (0.93-1.01) | 0.98 (0.94-1.03) |
| 4 | rs13107325 | 103188709 | T/C | T=0.080 | 1.07 (1.06-1.09) | 0.97 (0.90-1.04) | 1.01 (0.93-1.10) | 0.92 (0.84-1.01) |
| 5 | rs6450176 | 53298025 | A/G | A=0.260 | 1.03 (1.02-1.03) | 1.03 (0.99-1.07) | 1.03 (0.98-1.09) | 1.03 (0.97-1.08) |
| 6 | rs1936800 | 127436064 | T/C | C=0.490 | 1.02 (1.01-1.03) | 0.98 (0.95-1.02) | 0.99 (0.95-1.04) | 0.96 (0.91-1.00) |
| 6 | rs605066 | 139829666 | C/T | C=0.420 | 1.32 (1.20-1.46) | 1.05 (1.01-1.09) | 1.05 (1.00-1.09) | 1.06 (1.01-1.11) |
| 7 | rs702485 | 6449272 | A/G | G=0.450 | 1.02 (1.02-1.03) | 1.00 (0.96-1.03) | 1.01 (0.97-1.06) | 0.98 (0.94-1.03) |
| 7 | rs4142995 | 17919258 | T/G | T=0.380 | 1.03 (1.02-1.03) | 0.97 (0.94-1.01) | 0.96 (0.92-1.00) | 0.98 (0.93-1.02) |
| 7 | rs4917014 | 50305863 | T/G | G=0.320 | 1.02 (1.01-1.03) | 1.02 (0.98-1.06) | 1.01 (0.96-1.05) | 1.03 (0.98-1.08) |
| 7 | rs4731702 | 130433384 | C/T | T=0.490 | 1.03 (1.02-1.04) | 1.01 (0.97-1.04) | 1.00 (0.96-1.05) | 1.02 (0.97-1.07) |
| 8 | rs9987289 | 9183358 | A/G | A=0.100 | 2.27 (2.02-2.56) | 1.01 (0.95-1.08) | 1.04 (0.97-1.13) | 0.98 (0.90-1.07) |
| 8 | rs2293889 | 116599199 | T/G | T=0.410 | 1.03 (1.02-1.04) | 0.99 (0.94-1.04) | 1.00 (0.94-1.06) | 0.98 (0.92-1.05) |
| 9 | rs1883025 | 107664301 | T/C | T=0.250 | 1.07 (1.06-1.08) | 0.99 (0.95-1.03) | 0.95 (0.91-1.00) | 1.03 (0.98-1.09) |
| 10 | rs970548 | 46013277 | A/C | C=0.260 | 1.03 (1.02-1.03) | 1.00 (0.96-1.05) | 1.04 (0.98-1.09) | 0.97 (0.92-1.02) |
| 11 | rs12801636 | 65391317 | G/A | A=0.230 | 1.02 (1.02-1.03) | 0.98 (0.94-1.03) | 1.00 (0.95-1.05) | 0.96 (0.91-1.02) |
| 11 | rs499974 | 75455021 | A/C | A=0.190 | 1.03 (1.02-1.04) | 0.97 (0.93-1.02) | 0.96 (0.91-1.02) | 0.98 (0.92-1.04) |
| 11 | rs2923084 | 10388782 | G/A | G=0.180 | 1.03 (1.02-1.04) | 1.00 (0.95-1.04) | 1.00 (0.95-1.06) | 0.99 (0.93-1.05) |
| 11 | rs3136441 | 46743247 | T/C | C=0.180 | 1.06 (1.05-1.07) | 1.01 (0.96-1.07) | 1.05 (0.99-1.12) | 0.97 (0.91-1.04) |
| 12 | rs7134375 | 20473758 | C/A | A=0.430 | 1.02 (1.01-1.03) | 0.98 (0.94-1.01) | 0.98 (0.94-1.02) | 0.98 (0.93-1.02) |
| 12 | rs7134594 | 110000193 | C/T | C=0.480 | 1.42 (1.29-1.56) | 1.03 (0.99-1.07) | 1.00 (0.95-1.04) | 1.08 (1.03-1.14) |
| 12 | rs4759375 | 123796238 | C/T | T=0.080 | 1.06 (1.04-1.08) | 1.02 (0.96-1.09) | 1.05 (0.98-1.14) | 0.99 (0.92-1.08) |
| 12 | rs4765127 | 124460167 | G/T | T=0.350 | 1.03 (1.02-1.04) | 1.02 (0.98-1.06) | 1.00 (0.95-1.05) | 1.04 (0.99-1.09) |
| 12 | rs838880 | 125261593 | T/C | C=0.340 | 1.62 (1.49-1.75) | 0.99 (0.96-1.03) | 0.98 (0.94-1.03) | 1.01 (0.96-1.06) |
| 14 | rs4983559 | 105277209 | A/G | G=0.400 | 1.02 (1.01-1.03) | 1.05 (1.02-1.09) | 1.05 (1.00-1.09) | 1.06 (1.01-1.11) |
| 15 | rs1532085 | 58683366 | G/A | A=0.400 | 1.11 (1.10-1.12) | 1.02 (0.98-1.06) | 1.04 (1.00-1.09) | 1.00 (0.95-1.05) |
| 15 | rs2652834 | 63396867 | A/G | A=0.210 | 1.32 (1.22-1.44) | 0.99 (0.95-1.04) | 1.00 (0.95-1.06) | 0.97 (0.92-1.03) |
| 16 | rs1121980 | 53809247 | A/G | A=0.430 | 1.02 (1.01-1.03) | 1.02 (0.98-1.06) | 1.00 (0.96-1.05) | 1.04 (0.99-1.09) |
| 16 | rs3764261 | 56993324 | C/A | A=0.320 | 1.27 (1.26-1.28) | 0.99 (0.95-1.03) | 0.98 (0.94-1.03) | 1.00 (0.95-1.05) |
| 16 | rs16942887 | 67928042 | G/A | A=0.140 | 1.09 (1.08-1.10) | 1.00 (0.95-1.06) | 0.98 (0.92-1.05) | 1.04 (0.97-1.11) |
| 17 | rs11869286 | 37813856 | G/C | G=0.350 | 1.38 (1.28-1.48) | 0.99 (0.96-1.03) | 1.02 (0.97-1.06) | 0.97 (0.92-1.02) |
| 17 | rs4148008 | 66875294 | G/C | G=0.330 | 1.03 (1.02-1.04) | 1.00 (0.97-1.04) | 1.03 (0.98-1.08) | 0.99 (0.94-1.04) |
| 17 | rs4129767 | 76403984 | G/A | G=0.480 | 1.27 (1.19-1.36) | 0.99 (0.96-1.03) | 1.01 (0.96-1.05) | 0.97 (0.93-1.02) |
| 18 | rs7241918 | 47160953 | G/T | G=0.190 | 1.09 (1.08-1.11) | 1.03 (0.99-1.08) | 1.03 (0.97-1.09) | 1.05 (0.99-1.12) |
| 18 | rs12967135 | 57849023 | A/G | A=0.250 | 1.03 (1.02-1.04) | 0.97 (0.93-1.01) | 0.94 (0.90-0.99) | 0.99 (0.94-1.05) |
| 19 | rs17695224 | 52324216 | A/G | A=0.260 | 1.03 (1.02-1.04) | 1.00 (0.96-1.04) | 0.97 (0.92-1.02) | 1.04 (0.98-1.09) |
| 19 | rs7255436 | 8433196 | C/A | C=0.470 | 1.38 (1.23-1.54) | 1.01 (0.98-1.05) | 1.01 (0.96-1.05) | 1.02 (0.97-1.07) |
| 19 | rs737337 | 11347493 | C/T | C=0.110 | 1.06 (1.04-1.07) | 1.01 (0.95-1.07) | 1.06 (0.98-1.15) | 0.98 (0.90-1.06) |
| 19 | rs386000 | 54792761 | G/C | C=0.260 | 1.05 (1.04-1.06) | 1.00 (0.96-1.05) | 0.99 (0.94-1.05) | 1.02 (0.96-1.08) |
| 20 | rs1800961 | 43042364 | T/C | T=0.050 | 1.14 (1.11-1.16) | 1.03 (0.93-1.14) | 1.02 (0.90-1.16) | 1.06 (0.92-1.22) |
| 20 | rs6065906 | 44554015 | C/T | C=0.190 | 1.06 (1.05-1.07) | 1.05 (1.00-1.10) | 1.05 (0.99-1.11) | 1.04 (0.98-1.11) |
| 22 | rs181362 | 21932068 | T/C | T=0.230 | 1.04 (1.03-1.05) | 1.02 (0.98-1.07) | 1.03 (0.97-1.09) | 1.03 (0.97-1.09) |

LDL cholesterol

| **Chromosome** | **SNP** | **Base pair position *** | **Alleles**** | **MAF** | **LDL** | **All glioma** | **GBM** | **Non-GBM** |
| --- | --- | --- | --- | --- | --- | --- | --- | --- |
|  |  |  |  |  | **OR (95% CI)** | **OR (95% CI)** | **OR (95% CI)** | **OR (95% CI)** |
| 1 | rs2479409 | 55504650 | A/G | G=0.320 | 1.90 (1.74-2.06) | 0.98 (0.95-1.02) | 0.96 (0.92-1.01) | 1.02 (0.97-1.07) |
| 1 | rs629301 | 109818306 | G/T | G=0.240 | 1.18 (1.17-1.19) | 1.02 (0.98-1.06) | 1.01 (0.95-1.06) | 1.02 (0.96-1.08) |
| 2 | rs10490626 | 118835841 | A/G | A=0.080 | 1.05 (1.04-1.07) | 0.94 (0.88-1.01) | 0.97 (0.89-1.05) | 0.90 (0.83-0.98) |
| 2 | rs2030746 | 121309488 | C/T | T=0.400 | 1.02 (1.01-1.03) | 0.98 (0.94-1.01) | 0.97 (0.93-1.01) | 0.99 (0.94-1.04) |
| 2 | rs1367117 | 21263900 | G/A | A=0.320 | 1.13 (1.12-1.14) | 1.02 (0.99-1.06) | 0.99 (0.94-1.04) | 1.06 (1.00-1.11) |
| 2 | rs4299376 | 44072576 | T/G | G=0.310 | 1.08 (1.07-1.09) | 1.01 (0.97-1.05) | 1.02 (0.98-1.07) | 0.99 (0.95-1.05) |
| 3 | rs7640978 | 32533010 | T/C | T=0.090 | 1.04 (1.03-1.05) | 0.96 (0.90-1.02) | 0.96 (0.89-1.04) | 1.00 (0.92-1.09) |
| 3 | rs17404153 | 132163200 | T/G | T=0.140 | 1.03 (1.02-1.05) | 0.98 (0.93-1.04) | 0.97 (0.91-1.04) | 0.99 (0.92-1.07) |
| 5 | rs4530754 | 122855416 | G/A | G=0.460 | 1.03 (1.02-1.04) | 1.00 (0.97-1.04) | 1.00 (0.95-1.04) | 1.00 (0.96-1.05) |
| 6 | rs3757354 | 16127407 | T/C | T=0.240 | 1.04 (1.03-1.05) | 0.97 (0.93-1.01) | 0.96 (0.91-1.02) | 0.99 (0.93-1.05) |
| 6 | rs1800562 | 26093141 | A/G | A=0.070 | 1.06 (1.05-1.08) | 1.05 (0.98-1.13) | 1.06 (0.97-1.16) | 1.05 (0.95-1.16) |
| 6 | rs1564348 | 160578860 | T/C | C=0.180 | 1.05 (1.04-1.06) | 1.00 (0.96-1.05) | 1.01 (0.95-1.07) | 0.99 (0.93-1.06) |
| 7 | rs4722551 | 25991826 | T/C | C=0.200 | 1.04 (1.03-1.05) | 1.03 (0.98-1.08) | 1.03 (0.97-1.09) | 1.02 (0.96-1.09) |
| 8 | rs10102164 | 55421614 | G/A | A=0.210 | 1.03 (1.02-1.04) | 0.99 (0.95-1.04) | 0.97 (0.91-1.02) | 1.02 (0.96-1.08) |
| 8 | rs11136341 | 145043543 | A/G | G=0.400 | 1.05 (1.03-1.06) | 0.95 (0.90-1.01) | 0.96 (0.91-1.03) | 0.94 (0.87-1.00) |
| 9 | rs9411489 | 136155000 | C/T | T=0.210 | 1.08 (1.07-1.09) | 0.99 (0.96-1.03) | 0.96 (0.91-1.01) | 1.04 (0.98-1.10) |
| 11 | rs11220462 | 126243952 | G/A | A=0.140 | 1.06 (1.05-1.07) | 1.04 (0.99-1.10) | 1.02 (0.95-1.08) | 1.01 (0.94-1.08) |
| 13 | rs4942486 | 32953388 | C/T | T=0.480 | 1.02 (1.02-1.03) | 1.02 (0.99-1.06) | 1.05 (1.00-1.10) | 0.99 (0.94-1.03) |
| 14 | rs8017377 | 24883887 | G/A | A=0.460 | 1.03 (1.02-1.04) | 1.02 (0.98-1.06) | 1.03 (0.99-1.08) | 1.01 (0.96-1.05) |
| 17 | rs1801689 | 64210580 | A/C | C=0.040 | 1.11 (1.08-1.14) | 0.93 (0.84-1.04) | 0.94 (0.82-1.07) | 0.94 (0.82-1.08) |
| 19 | rs6511720 | 11202306 | T/G | T=0.120 | 1.25 (1.23-1.26) | 1.03 (0.97-1.08) | 0.99 (0.92-1.06) | 1.07 (1.00-1.15) |
| 19 | rs4420638 | 45422946 | A/G | G=0.190 | 1.25 (1.23-1.27) | 1.02 (0.97-1.07) | 1.03 (0.97-1.09) | 1.00 (0.94-1.07) |
| 20 | rs364585 | 12962718 | A/G | A=0.380 | 1.03 (1.02-1.03) | 1.03 (0.99-1.07) | 1.04 (0.99-1.08) | 1.02 (0.98-1.08) |
| 20 | rs2328223 | 17845921 | A/C | C=0.210 | 1.03 (1.02-1.04) | 0.98 (0.94-1.03) | 0.94 (0.89-0.99) | 1.03 (0.97-1.09) |
| 20 | rs6029526 | 39672618 | T/A | A=0.470 | 1.04 (1.03-1.06) | 0.98 (0.95-1.02) | 1.00 (0.96-1.04) | 0.97 (0.92-1.01) |
| 22 | rs5763662 | 30378703 | C/T | T=0.040 | 1.08 (1.05-1.11) | 1.06 (0.94-1.21) | 1.05 (0.90-1.22) | 1.05 (0.89-1.24) |

Type-2 diabetes

| **Chromosome** | **SNP** | **Base pair position *** | **Alleles**** | **MAF** | **Type-2 diabetes** | **All glioma** | **GBM** | **Non-GBM** |
| --- | --- | --- | --- | --- | --- | --- | --- | --- |
|  |  |  |  |  | **OR (95% CI)** | **OR (95% CI)** | **OR (95% CI)** | **OR (95% CI)** |
| 1 | rs340874 | 214159256 | T/C | T=0.480 | 1.07 (1.05-1.09) | 1.00 (0.97-1.04) | 0.99 (0.94-1.03) | 1.02 (0.97-1.07) |
| 2 | rs780094 | 27741237 | T/C | T=0.389 | 1.06 (1.04-1.08) | 1.04 (1.01-1.08) | 1.07 (1.02-1.11) | 1.02 (0.97-1.07) |
| 2 | rs77981966 | 43777964 | T/C | T=0.066 | 1.16 (1.12-1.21) | 1.05 (0.98-1.12) | 1.04 (0.95-1.13) | 1.08 (0.99-1.19) |
| 2 | rs243020 | 60585028 | A/G | A=0.462 | 1.06 (1.04-1.08) | 0.98 (0.95-1.02) | 0.96 (0.92-1.00) | 1.01 (0.97-1.06) |
| 2 | rs75297654 | 165545615 | T/C | C=0.124 | 1.11 (1.09-1.13) | 1.04 (0.99-1.10) | 1.02 (0.96-1.09) | 1.07 (0.99-1.14) |
| 3 | rs11712037 | 12344730 | G/C | C=0.138 | 1.14 (1.11-1.17) | 1.01 (0.96-1.07) | 1.00 (0.93-1.06) | 1.06 (0.98-1.14) |
| 3 | rs17676309 | 64730121 | T/C | C=0.410 | 1.07 (1.05-1.09) | 1.02 (0.98-1.05) | 1.02 (0.97-1.07) | 1.01 (0.96-1.06) |
| 3 | rs11708067 | 123065778 | G/A | A=0.214 | 1.11 (1.09-1.13) | 1.02 (0.97-1.06) | 1.01 (0.96-1.07) | 0.99 (0.94-1.05) |
| 3 | rs35510946 | 185518910 | G/A | G=0.301 | 1.14 (1.12-1.16) | 0.99 (0.96-1.03) | 0.99 (0.95-1.04) | 0.99 (0.94-1.04) |
| 4 | rs10937721 | 6306763 | G/C | C=0.420 | 1.09 (1.07-1.11) | 0.98 (0.94-1.01) | 0.96 (0.92-1.01) | 0.98 (0.93-1.03) |
| 5 | rs7732130 | 76435004 | A/G | A=0.278 | 1.08 (1.06-1.10) | 1.06 (1.02-1.10) | 1.05 (1.00-1.10) | 1.09 (1.04-1.15) |
| 6 | rs35261542 | 20675792 | C/A | C=0.280 | 1.17 (1.15-1.19) | 0.97 (0.93-1.01) | 0.94 (0.90-0.99) | 1.01 (0.96-1.07) |
| 7 | rs10276674 | 14922007 | T/C | T=0.183 | 1.08 (1.06-1.10) | 1.02 (0.96-1.09) | 1.00 (0.93-1.08) | 1.04 (0.95-1.13) |
| 7 | rs1974620 | 15065467 | C/T | T=0.481 | 1.06 (1.04-1.08) | 1.01 (0.98-1.05) | 1.01 (0.97-1.06) | 1.01 (0.96-1.05) |
| 7 | rs1513272 | 28200097 | T/C | C=0.482 | 1.10 (1.08-1.12) | 0.99 (0.96-1.03) | 1.01 (0.97-1.06) | 0.97 (0.93-1.02) |
| 7 | rs878521 | 44255643 | G/A | G=0.235 | 1.07 (1.05-1.09) | 0.97 (0.93-1.01) | 0.99 (0.94-1.04) | 0.95 (0.90-1.01) |
| 8 | rs13266634 | 118184783 | T/C | C=0.324 | 1.12 (1.10-1.14) | 0.99 (0.95-1.03) | 0.98 (0.93-1.02) | 1.00 (0.95-1.05) |
| 9 | rs10811660 | 22134068 | A/G | G=0.170 | 1.27 (1.23-1.31) | 0.98 (0.94-1.03) | 0.97 (0.92-1.03) | 0.99 (0.93-1.05) |
| 10 | rs11187140 | 94466910 | A/G | G=0.368 | 1.12 (1.10-1.14) | 1.01 (0.97-1.05) | 1.02 (0.97-1.06) | 1.00 (0.96-1.06) |
| 10 | rs7903146 | 114758349 | C/T | C=0.260 | 1.36 (1.33-1.39) | 1.01 (0.97-1.05) | 1.01 (0.96-1.06) | 1.01 (0.96-1.06) |
| 11 | rs2237895 | 2857194 | A/C | A=0.428 | 1.07 (1.05-1.09) | 0.99 (0.95-1.03) | 0.98 (0.93-1.02) | 1.00 (0.95-1.05) |
| 11 | rs5215 | 17408630 | T/C | T=0.389 | 1.07 (1.05-1.09) | 1.02 (0.98-1.06) | 1.02 (0.98-1.07) | 0.99 (0.95-1.04) |
| 11 | rs142489578 | 72460930 | A/AT | AT=0.177 | 1.10 (1.07-1.13) | 1.03 (0.92-1.14) | 0.97 (0.85-1.11) | 1.15 (1.00-1.33) |
| 11 | rs10830963 | 92708710 | C/G | C=0.283 | 1.09 (1.07-1.11) | 1.02 (0.97-1.06) | 0.99 (0.94-1.04) | 1.03 (0.98-1.09) |
| 12 | rs2583941 | 66204598 | G/A | G=0.090 | 1.11 (1.08-1.14) | 1.00 (0.92-1.09) | 1.02 (0.93-1.13) | 0.98 (0.88-1.09) |
| 12 | rs1169288 | 121416650 | A/C | A=0.334 | 1.09 (1.07-1.11) | 1.02 (0.98-1.06) | 1.01 (0.96-1.06) | 1.01 (0.96-1.07) |
| 12 | rs1800574 | 121416864 | C/T | C=0.027 | 1.22 (1.15-1.30) | 1.10 (0.98-1.24) | 1.01 (0.87-1.17) | 1.17 (0.99-1.37) |
| 15 | rs7161785 | 62395224 | C/G | G=0.445 | 1.06 (1.04-1.08) | 1.00 (0.96-1.04) | 0.97 (0.93-1.02) | 1.05 (1.00-1.10) |
| 15 | rs3803563 | 91531352 | C/A | C=0.175 | 1.08 (1.06-1.11) | 1.02 (0.98-1.07) | 1.04 (0.99-1.10) | 1.02 (0.96-1.08) |
| 16 | rs9927317 | 53820996 | C/G | C=0.396 | 1.14 (1.12-1.16) | 0.99 (0.94-1.04) | 1.00 (0.94-1.06) | 0.99 (0.93-1.06) |
| 17 | rs4430796 | 36098040 | A/G | A=0.455 | 1.09 (1.07-1.11) | 1.02 (0.98-1.06) | 1.02 (0.97-1.07) | 1.02 (0.97-1.07) |
| 19 | rs72999033 | 19366632 | C/T | C=0.069 | 1.16 (1.12-1.20) | 0.99 (0.92-1.06) | 1.02 (0.93-1.11) | 0.97 (0.88-1.06) |
| 19 | rs2238689 | 46178661 | T/C | T=0.425 | 1.08 (1.06-1.10) | 1.03 (1.00-1.07) | 1.03 (0.99-1.08) | 1.05 (1.00-1.10) |
| 20 | rs1800961 | 43042364 | C/T | C=0.034 | 1.16 (1.10-1.22) | 0.97 (0.88-1.08) | 0.98 (0.86-1.11) | 0.95 (0.82-1.09) |

Total cholesterol

| **Chromosome** | **SNP** | **Base pair position *** | **Alleles**** | **MAF** | **Total cholesterol** | **All glioma** | **GBM** | **Non-GBM** |
| --- | --- | --- | --- | --- | --- | --- | --- | --- |
|  |  |  |  |  | **OR (95% CI)** | **OR (95% CI)** | **OR (95% CI)** | **OR (95% CI)** |
| 1 | rs1077514 | 23766233 | C/T | C=0.150 | 1.03 (1.02-1.04) | 0.99 (0.94-1.05) | 1.01 (0.95-1.08) | 0.99 (0.93-1.06) |
| 1 | rs12027135 | 25775733 | C/T | A=0.460 | 1.03 (1.02-1.04) | 1.00 (0.97-1.04) | 0.99 (0.95-1.03) | 1.01 (0.97-1.06) |
| 1 | rs7515577 | 93009438 | C/A | C=0.230 | 1.04 (1.02-1.05) | 1.04 (1.00-1.09) | 1.05 (1.00-1.11) | 1.03 (0.97-1.09) |
| 1 | rs2642442 | 220973563 | C/T | C=0.330 | 1.04 (1.02-1.05) | 1.00 (0.96-1.04) | 0.98 (0.94-1.03) | 1.01 (0.96-1.06) |
| 1 | rs514230 | 234858597 | C/T | A=0.480 | 1.04 (1.03-1.05) | 1.01 (0.97-1.04) | 1.00 (0.96-1.05) | 1.02 (0.98-1.07) |
| 2 | rs2287623 | 169830155 | A/G | G=0.410 | 1.03 (1.02-1.04) | 1.00 (0.97-1.04) | 0.98 (0.94-1.03) | 1.03 (0.99-1.09) |
| 2 | rs11694172 | 203532304 | C/G | G=0.250 | 1.03 (1.02-1.04) | 0.99 (0.95-1.04) | 1.00 (0.95-1.05) | 0.98 (0.93-1.04) |
| 2 | rs11563251 | 234679384 | C/T | T=0.120 | 1.04 (1.03-1.05) | 1.01 (0.95-1.07) | 1.00 (0.93-1.07) | 1.01 (0.94-1.09) |
| 3 | rs13315871 | 58381287 | A/G | A=0.100 | 1.04 (1.02-1.05) | 1.04 (0.98-1.11) | 1.03 (0.96-1.11) | 1.02 (0.94-1.11) |
| 3 | rs2290159 | 12628920 | C/G | C=0.230 | 1.04 (1.03-1.05) | 1.02 (0.98-1.07) | 1.03 (0.97-1.08) | 1.03 (0.97-1.09) |
| 5 | rs12916 | 74656539 | C/C | C=0.400 | 1.07 (1.06-1.08) | 1.00 (0.97-1.04) | 0.99 (0.94-1.03) | 1.02 (0.97-1.07) |
| 5 | rs6882076 | 156390297 | C/C | T=0.360 | 1.05 (1.04-1.06) | 1.00 (0.96-1.04) | 0.98 (0.93-1.02) | 1.02 (0.97-1.07) |
| 6 | rs2758886 | 39250837 | C/A | A=0.300 | 1.02 (1.01-1.03) | 0.99 (0.95-1.03) | 0.97 (0.93-1.02) | 1.01 (0.96-1.06) |
| 6 | rs9376090 | 135411228 | C/C | T=0.280 | 1.03 (1.02-1.03) | 1.03 (0.98-1.07) | 1.06 (1.00-1.11) | 0.98 (0.93-1.04) |
| 6 | rs3177928 | 32412435 | C/A | A=0.170 | 1.05 (1.04-1.06) | 1.02 (0.97-1.08) | 1.02 (0.96-1.09) | 1.03 (0.96-1.11) |
| 6 | rs2814982 | 34546560 | T/C | T=0.120 | 1.55 (1.39-1.73) | 0.97 (0.92-1.03) | 0.99 (0.92-1.07) | 0.94 (0.87-1.01) |
| 6 | rs9488822 | 116312893 | C/T | T=0.360 | 1.03 (1.02-1.05) | 1.01 (0.98-1.05) | 1.00 (0.96-1.05) | 1.02 (0.97-1.07) |
| 7 | rs1997243 | 1083777 | C/G | G=0.130 | 1.03 (1.02-1.04) | 1.04 (0.98-1.09) | 1.05 (0.99-1.12) | 1.02 (0.95-1.09) |
| 7 | rs12670798 | 21607352 | C/C | C=0.250 | 1.04 (1.03-1.05) | 1.02 (0.97-1.06) | 1.04 (0.98-1.09) | 1.00 (0.94-1.06) |
| 7 | rs2072183 | 44579180 | C/C | C=0.290 | 1.04 (1.03-1.05) | 1.02 (0.97-1.06) | 1.01 (0.96-1.07) | 1.01 (0.95-1.07) |
| 8 | rs2081687 | 59388565 | C/T | T=0.360 | 1.04 (1.03-1.05) | 1.01 (0.97-1.05) | 1.03 (0.98-1.08) | 0.99 (0.95-1.04) |
| 9 | rs3780181 | 2640759 | G/A | G=0.080 | 1.04 (1.03-1.06) | 0.99 (0.93-1.07) | 1.00 (0.92-1.09) | 0.96 (0.87-1.05) |
| 10 | rs10904908 | 17260290 | C/G | G=0.430 | 1.03 (1.02-1.03) | 0.97 (0.94-1.01) | 0.98 (0.94-1.02) | 0.98 (0.94-1.03) |
| 10 | rs2255141 | 113933886 | G/A | A=0.300 | 1.03 (1.02-1.04) | 1.01 (0.96-1.06) | 1.01 (0.95-1.07) | 1.03 (0.96-1.10) |
| 11 | rs10128711 | 18632984 | C/C | T=0.300 | 1.03 (1.02-1.04) | 0.96 (0.92-1.00) | 0.95 (0.91-1.00) | 0.98 (0.93-1.03) |
| 11 | rs7941030 | 122522375 | C/C | C=0.390 | 1.03 (1.02-1.04) | 0.99 (0.95-1.02) | 0.99 (0.94-1.03) | 0.98 (0.94-1.03) |
| 12 | rs4883201 | 9082581 | G/A | G=0.120 | 1.04 (1.02-1.05) | 0.94 (0.89-1.00) | 0.94 (0.88-1.01) | 0.93 (0.86-1.01) |
| 12 | rs11065987 | 112072424 | G/A | G=0.410 | 1.03 (1.02-1.04) | 1.01 (0.98-1.05) | 1.03 (0.99-1.08) | 1.01 (0.96-1.06) |
| 12 | rs1169288 | 121416650 | C/C | C=0.340 | 1.03 (1.02-1.04) | 1.02 (0.98-1.06) | 1.01 (0.96-1.06) | 1.01 (0.96-1.07) |
| 16 | rs2000999 | 72108093 | C/A | A=0.200 | 1.06 (1.05-1.07) | 1.01 (0.96-1.05) | 1.00 (0.94-1.05) | 1.01 (0.95-1.07) |
| 17 | rs314253 | 7091650 | C/T | C=0.370 | 1.02 (1.02-1.03) | 0.99 (0.95-1.02) | 1.00 (0.96-1.05) | 0.96 (0.91-1.01) |
| 19 | rs10401969 | 19407718 | C/T | C=0.090 | 1.15 (1.13-1.16) | 1.01 (0.95-1.08) | 0.98 (0.91-1.07) | 1.04 (0.96-1.14) |
| 19 | rs492602 | 49206417 | C/G | T=0.470 | 1.03 (1.02-1.04) | 0.99 (0.95-1.03) | 0.98 (0.94-1.03) | 0.98 (0.93-1.03) |
| 20 | rs2277862 | 34152782 | T/C | T=0.150 | 1.04 (1.02-1.05) | 1.02 (0.97-1.08) | 1.07 (1.00-1.13) | 0.99 (0.93-1.06) |
| 20 | rs2902940 | 39091487 | G/A | G=0.300 | 1.02 (1.02-1.03) | 0.98 (0.94-1.02) | 0.98 (0.93-1.03) | 0.97 (0.92-1.02) |
| 22 | rs138777 | 35711098 | G/A | A=0.360 | 1.02 (1.01-1.03) | 0.97 (0.94-1.01) | 0.98 (0.93-1.02) | 0.97 (0.92-1.02) |
| 22 | rs4253772 | 46627603 | C/T | T=0.110 | 1.03 (1.02-1.04) | 1.00 (0.94-1.05) | 0.99 (0.93-1.07) | 1.00 (0.93-1.08) |

Triglycerides

| **Chromosome** | **SNP** | **Base pair position *** | **Alleles**** | **MAF** | **Triglycerides** | **All glioma** | **GBM** | **Non-GBM** |
| --- | --- | --- | --- | --- | --- | --- | --- | --- |
|  |  |  |  |  | **OR (95% CI)** | **OR (95% CI)** | **OR (95% CI)** | **OR (95% CI)** |
| 1 | rs2131925 | 63025942 | G/T | G=0.340 | 1.93 (1.80-2.08) | 1.06 (1.02-1.10) | 1.03 (0.98-1.08) | 1.10 (1.04-1.15) |
| 2 | rs1260326 | 27730940 | C/T | T=0.390 | 1.12 (1.11-1.13) | 0.96 (0.92-0.99) | 0.93 (0.89-0.98) | 0.99 (0.94-1.04) |
| 3 | rs645040 | 135926622 | G/T | G=0.230 | 1.34 (1.23-1.45) | 0.97 (0.93-1.01) | 0.95 (0.90-1.00) | 0.99 (0.93-1.04) |
| 4 | rs6831256 | 3473139 | A/G | G=0.420 | 1.03 (1.02-1.03) | 0.96 (0.92-0.99) | 0.95 (0.91-0.99) | 0.98 (0.93-1.02) |
| 4 | rs442177 | 88030261 | G/T | G=0.420 | 1.03 (1.02-1.04) | 0.99 (0.96-1.03) | 1.01 (0.97-1.06) | 0.97 (0.92-1.01) |
| 5 | rs9686661 | 55861786 | C/T | T=0.200 | 1.46 (1.33-1.60) | 0.95 (0.91-1.00) | 0.95 (0.90-1.00) | 0.97 (0.92-1.03) |
| 6 | rs998584 | 43757896 | C/A | A=0.490 | 1.03 (1.02-1.04) | 1.01 (0.97-1.05) | 0.99 (0.95-1.03) | 1.03 (0.98-1.08) |
| 7 | rs38855 | 116358044 | G/A | G=0.470 | 1.02 (1.01-1.03) | 1.01 (0.98-1.05) | 1.01 (0.96-1.05) | 1.01 (0.97-1.06) |
| 7 | rs17145738 | 72982874 | T/C | T=0.130 | 1.12 (1.11-1.13) | 0.97 (0.92-1.03) | 0.99 (0.93-1.06) | 0.94 (0.87-1.01) |
| 8 | rs11776767 | 10683929 | G/C | C=0.370 | 1.02 (1.02-1.03) | 1.00 (0.96-1.03) | 1.01 (0.96-1.05) | 0.99 (0.95-1.04) |
| 8 | rs1495741 | 18272881 | A/G | G=0.260 | 1.04 (1.03-1.05) | 1.00 (0.96-1.04) | 0.99 (0.94-1.05) | 0.99 (0.94-1.05) |
| 8 | rs12678919 | 19844222 | G/A | G=0.130 | 1.19 (1.17-1.20) | 0.99 (0.94-1.05) | 0.95 (0.89-1.03) | 1.02 (0.95-1.11) |
| 8 | rs2954029 | 126490972 | T/A | T=0.470 | 1.08 (1.07-1.09) | 0.97 (0.94-1.01) | 0.96 (0.92-1.00) | 0.99 (0.95-1.04) |
| 10 | rs1832007 | 5254847 | G/A | G=0.180 | 1.03 (1.02-1.04) | 1.03 (0.98-1.08) | 1.01 (0.95-1.07) | 1.04 (0.98-1.12) |
| 10 | rs10761731 | 65027610 | T/A | T=0.440 | 1.03 (1.02-1.04) | 1.02 (0.98-1.06) | 1.05 (1.00-1.10) | 1.01 (0.96-1.06) |
| 10 | rs2068888 | 94839642 | A/G | A=0.450 | 1.02 (1.02-1.03) | 0.98 (0.95-1.02) | 0.98 (0.94-1.03) | 0.96 (0.92-1.01) |
| 11 | rs174546 | 61569830 | C/T | T=0.360 | 1.05 (1.04-1.05) | 1.01 (0.97-1.05) | 1.00 (0.96-1.05) | 1.03 (0.98-1.08) |
| 11 | rs964184 | 116648917 | C/G | G=0.160 | 1.26 (1.25-1.28) | 1.01 (0.96-1.07) | 1.02 (0.96-1.09) | 0.99 (0.92-1.06) |
| 12 | rs11613352 | 57792580 | T/C | T=0.260 | 1.03 (1.02-1.04) | 0.99 (0.95-1.03) | 0.98 (0.93-1.03) | 1.00 (0.95-1.06) |
| 15 | rs2412710 | 42683787 | G/A | A=0.040 | 1.10 (1.07-1.14) | 1.05 (0.93-1.19) | 1.09 (0.93-1.27) | 1.04 (0.88-1.22) |
| 16 | rs3198697 | 15129940 | T/C | T=0.430 | 1.02 (1.01-1.03) | 0.99 (0.96-1.03) | 1.01 (0.96-1.05) | 0.97 (0.93-1.02) |
| 17 | rs8077889 | 41878166 | A/C | C=0.220 | 1.03 (1.02-1.03) | 0.96 (0.91-1.00) | 0.96 (0.91-1.02) | 0.95 (0.90-1.01) |
| 19 | rs7248104 | 7224431 | A/G | A=0.420 | 1.02 (1.02-1.03) | 1.00 (0.96-1.04) | 0.98 (0.93-1.04) | 0.99 (0.94-1.05) |
| 19 | rs731839 | 33899065 | A/G | G=0.350 | 1.02 (1.01-1.03) | 1.00 (0.96-1.03) | 0.98 (0.94-1.03) | 1.02 (0.97-1.07) |

WHR

| **Chromosome** | **SNP** | **Position*** | **Alleles**** | **MAF** | **WHR** | **All glioma** | **GBM** | **Non-GBM** |
| --- | --- | --- | --- | --- | --- | --- | --- | --- |
|  |  |  |  |  | **OR (95% CI)** | **OR (95% CI)** | **OR (95% CI)** | **OR (95% CI)** |
| 1 | rs2765539 | 119549418 | C/T | C=0.292 | 1.03 (1.02-1.04) | 1.00 (0.95-1.06) | 0.98 (0.92-1.04) | 1.02 (0.96-1.10) |
| 1 | rs1011731 | 172346548 | A/G | G=0.458 | 1.02 (1.01-1.03) | 0.98 (0.94-1.01) | 0.98 (0.93-1.02) | 0.99 (0.95-1.04) |
| 1 | rs1563355 | 219653101 | T/C | T=0.314 | 1.03 (1.02-1.04) | 1.02 (0.98-1.06) | 0.99 (0.95-1.04) | 1.05 (1.00-1.10) |
| 2 | rs929641 | 58792377 | G/A | G=0.383 | 1.02 (1.01-1.03) | 1.02 (0.99-1.06) | 1.00 (0.96-1.05) | 1.04 (0.99-1.09) |
| 2 | rs1128249 | 165528624 | T/G | T=0.442 | 1.02 (1.01-1.03) | 1.04 (1.00-1.07) | 1.02 (0.98-1.07) | 1.04 (1.00-1.09) |
| 2 | rs1569135 | 188115398 | G/A | G=0.467 | 1.02 (1.02-1.03) | 0.98 (0.95-1.01) | 0.97 (0.93-1.01) | 0.98 (0.93-1.03) |
| 3 | rs2972164 | 12334416 | T/C | T=0.500 | 1.02 (1.01-1.03) | 1.03 (0.99-1.07) | 1.03 (0.99-1.08) | 1.03 (0.98-1.08) |
| 3 | rs9860730 | 64701146 | G/A | G=0.233 | 1.02 (1.02-1.03) | 0.99 (0.96-1.03) | 1.01 (0.96-1.06) | 0.97 (0.93-1.02) |
| 3 | rs17451107 | 156797609 | C/T | C=0.375 | 1.02 (1.02-1.03) | 0.97 (0.94-1.01) | 0.97 (0.93-1.02) | 0.99 (0.94-1.04) |
| 5 | rs459193 | 55806751 | G/A | A=0.217 | 1.03 (1.02-1.03) | 1.01 (0.97-1.05) | 1.02 (0.97-1.07) | 1.01 (0.95-1.06) |
| 6 | rs1294421 | 6743149 | T/G | T=0.400 | 1.03 (1.02-1.03) | 1.01 (0.97-1.05) | 1.01 (0.97-1.06) | 1.00 (0.96-1.05) |
| 6 | rs11755724 | 7118990 | A/G | A=0.300 | 1.02 (1.01-1.03) | 0.98 (0.95-1.02) | 0.97 (0.92-1.02) | 0.99 (0.94-1.04) |
| 6 | rs998584 | 43757896 | C/A | A=0.475 | 1.03 (1.02-1.04) | 1.01 (0.97-1.05) | 0.99 (0.95-1.03) | 1.03 (0.98-1.08) |
| 6 | rs2207139 | 50845490 | A/G | G=0.100 | 1.03 (1.02-1.03) | 0.99 (0.95-1.04) | 0.97 (0.91-1.03) | 1.02 (0.96-1.08) |
| 6 | rs9491696 | 127452639 | C/G | C=0.475 | 1.04 (1.03-1.05) | 1.03 (1.00-1.07) | 1.02 (0.98-1.07) | 1.05 (1.00-1.10) |
| 7 | rs10245353 | 25858614 | C/A | A=0.183 | 1.03 (1.02-1.04) | 1.02 (0.97-1.07) | 1.02 (0.96-1.08) | 1.04 (0.98-1.10) |
| 7 | rs7801581 | 27223771 | C/T | T=0.258 | 1.02 (1.01-1.03) | 1.03 (0.99-1.08) | 1.04 (0.99-1.10) | 1.05 (0.99-1.11) |
| 8 | rs12549058 | 72492238 | T/G | G=0.058 | 1.04 (1.03-1.05) | 0.94 (0.87-1.01) | 1.00 (0.92-1.10) | 0.87 (0.79-0.96) |
| 11 | rs4929927 | 8658485 | A/G | A=0.275 | 1.02 (1.01-1.03) | 1.04 (1.00-1.08) | 1.03 (0.98-1.08) | 1.04 (0.99-1.10) |
| 12 | rs11048470 | 26487283 | G/T | T=0.233 | 1.03 (1.02-1.03) | 0.99 (0.95-1.03) | 0.98 (0.94-1.03) | 1.01 (0.95-1.06) |
| 12 | rs10783615 | 54349773 | A/G | G=0.133 | 1.04 (1.03-1.05) | 1.02 (0.97-1.08) | 1.01 (0.95-1.08) | 1.03 (0.96-1.11) |
| 12 | rs10876528 | 54421476 | C/A | A=0.310 | 1.03 (1.02-1.04) | 1.01 (0.97-1.05) | 1.00 (0.95-1.04) | 1.03 (0.98-1.08) |
| 12 | rs1316952 | 124399550 | C/T | C=0.117 | 1.03 (1.02-1.04) | 0.98 (0.93-1.03) | 0.98 (0.92-1.04) | 0.97 (0.91-1.04) |
| 14 | rs17109256 | 79939993 | G/A | A=0.275 | 1.02 (1.02-1.03) | 1.04 (1.00-1.09) | 1.01 (0.95-1.06) | 1.09 (1.03-1.16) |
| 15 | rs1440372 | 67033151 | T/C | T=0.258 | 1.02 (1.01-1.03) | 0.99 (0.96-1.03) | 1.01 (0.96-1.06) | 0.97 (0.92-1.02) |
| 16 | rs1121980 | 53809247 | G/A | A=0.475 | 1.04 (1.04-1.05) | 0.98 (0.95-1.02) | 1.00 (0.96-1.04) | 0.96 (0.92-1.01) |
| 17 | rs4640244 | 21284223 | A/G | G=0.375 | 1.02 (1.01-1.03) | 0.99 (0.95-1.03) | 0.99 (0.94-1.03) | 0.99 (0.94-1.04) |
| 18 | rs11663816 | 57876227 | T/C | C=0.317 | 1.03 (1.02-1.03) | 1.02 (0.98-1.06) | 1.03 (0.98-1.08) | 1.00 (0.95-1.05) |
| 19 | rs3786897 | 33893008 | A/G | G=0.408 | 1.02 (1.02-1.03) | 1.04 (1.00-1.08) | 1.04 (0.99-1.08) | 1.04 (0.99-1.09) |
| 19 | rs2075650 | 45395619 | G/A | G=0.142 | 1.03 (1.02-1.04) | 0.95 (0.90-1.00) | 0.94 (0.88-1.00) | 0.96 (0.90-1.03) |
| 19 | rs2287019 | 46202172 | T/C | T=0.150 | 1.03 (1.02-1.04) | 0.96 (0.91-1.00) | 0.96 (0.91-1.02) | 0.94 (0.89-1.00) |
| 20 | rs16996700 | 50981945 | C/T | C=0.300 | 1.02 (1.01-1.03) | 0.99 (0.95-1.03) | 0.98 (0.93-1.02) | 1.01 (0.96-1.06) |
| 22 | rs2179129 | 29450923 | G/A | G=0.450 | 1.02 (1.01-1.03) | 1.00 (0.96-1.04) | 0.99 (0.95-1.04) | 1.00 (0.96-1.05) |

* NCBI build 37; ** Reference allele/effect allele; ORs are measured per unit of SD increment. BMI, body mass index; CI, confidence interval; GBM, glioblastoma multiforme; GSMR, generalised summary-data-based Mendelian randomisation; HDL, high-density lipoprotein; IV, instrumental variable; LDL, low-density lipoprotein; MAF, minor allele frequency; OR, odds ratio; SD, standard deviation; WHR, waist-hip ratio.
