# Supplementary material for: Influence of obesity-related risk factors in the aetiology of glioma
Source: Br J Cancer. 2018 Mar 13;118(7):1020–7. doi: 10.1038/s41416-018-0009-x (PMC5931112; doi:10.1038/s41416-018-0009-x)
Supplement: Supplementary file 3 — Supplementary Table 3(DOCX 17 kb) [file 41416_2018_9_MOESM3_ESM.docx]

| **Series** | **Study centre** | **Sampling** | **Cases** | **Controls** |
| --- | --- | --- | --- | --- |
| FRE | Groupe Hospitalier Pitié-Salpêtrière Paris | Patients with glioma were ascertained through the Service de Neurologie Mazarin, Groupe Hospitalier Pitié-Salpêtrière Paris. Controls were ascertained from the SU.VI.MAX (Supplementation en Vitamines et MinerauxAntioXydants) study. | 1423 | 1190 |
| GER | University of Bonn | Comprised of patients who had undergone surgery for a glioma at the Department of Neurosurgery, University of Bonn Medical Center, between 1996 and 2008. Control subjects were taken from three population studies: KORA (Co- operative Health Research in the Region of Augsburg); POPGEN (Population Genetic Cohort) and the Heinz Nixdorf Recall study. | 846 | 1310 |
| GICC | GLIOGENE Consortium | Comprise glioma cases and controls that were ascertained through Brigham and Women's Hospital (Boston, Massachusetts), Case Western Reserve University (Cleveland, Ohio), Columbia University (New York, New York), the Danish Cancer Society Research Centre (Copenhagen, Denmark), the Gertner Institute (Tel Hashomer, Israel), Duke University (Durham, North Carolina), the University of Texas MD Anderson Cancer Center (Houston, Texas), Memorial Sloan Kettering Cancer Center (New York, New York), the Mayo Clinic (Rochester, Minnesota), NorthShore HealthSystem (Chicago, Illinois), Umeå University (Umeå, Sweden), the University of California, San Francisco (San Francisco, California), the University of Southern California (Los Angeles, California), and the Institute of Cancer Research (London, United Kingdom). Cases had newly diagnosed glioma, and controls had no personal history of central nervous system tumor at the time of ascertainment | 4564 | 3265 |
| MDA | The University of Texas M.D. Anderson Cancer Center | Cases were ascertained through the MD Anderson Cancer Center, Texas, between 1990 and 2008. Individuals from the Cancer Genetic Markers of Susceptibility studies served as controls. | 1175 | 2236 |
| GiomaScan (NIH) | National Cancer Institute | Cases were newly diagnosed glioma (ICDO-3 codes 9380-9480 or equivalent), and controls were cancer-free at the time of glioma diagnosis. | 1653 | 2725 |
| UCSF-Mayo | Mayo Clinic | Comprised of Mayo cases, UCSF cases, and Mayo Clinic Biobank control data | 1519 | 804 |
| UCSF (SFAGS) | University of California, San Francisco | Cases were adults with newly diagnosed, histologically confirmed glioma. Population-based cases who were diagnosed between 1991 and 2009 and who were residing in the six San Francisco Bay area counties were ascertained using the Cancer Prevention Institute of California's early-case ascertainment system. Clinic-based cases who were diagnosed between 2002 and 2012 were recruited from the UCSF Neuro-oncology Clinic, regardless of the place of residence. From 1991 to 2010, population-based controls from the same residential area as the population-based cases were identified using random digit-dialing and were frequency matched to population-based cases for age, gender and ethnicity. Between 2010 and 2012, all controls were selected from the UCSF general medicine phlebotomy clinic. Clinic-based controls were matched to clinic-based glioma cases for age, gender and ethnicity. | 677 | 3940 |
| UK | INTERPHONE | Cases were ascertained through the INTERPHONE study. Individuals from the 1958 Birth Cohort served as a source of controls. | 631 | 2699 |
| Total |  |  | 12488 | 18169 |

**Supplementary Table 3: Summary of the eight glioma genome wide association studies (GWAS).**
