# Supplementary material for: Influence of obesity-related risk factors in the aetiology of glioma
Source: Br J Cancer. 2018 Mar 13;118(7):1020–7. doi: 10.1038/s41416-018-0009-x (PMC5931112; doi:10.1038/s41416-018-0009-x)
Supplement: Supplementary file 4 — Supplementary Tables Legends(DOCX 13 kb) [file 41416_2018_9_MOESM4_ESM.docx]

**Supplementary Table 1: Effect allele, frequencies, effect on each obesity-related trait and strength of association with glioma for SNPs used as genetic instruments.**

BMI, body mass index; CI, confidence interval; GBM, glioblastoma multiforme; GSMR, generalised summary-data-based Mendelian randomisation; HDL, high-density lipoprotein; IV, instrumental variable; LDL, low-density lipoprotein; MAF, minor allele frequency; OR, odds ratio; SD, standard deviation; WHR, waist-hip ratio.

**Supplementary Table 2: Pairwise *r*^2^ calculations for the SNPs used as genetic instruments.**

BMI, body mass index; HDL, high-density lipoprotein; LDL, low-density lipoprotein; WHR, waist-hip ratio.

**Supplementary Table 3: Summary of the eight glioma genome wide association studies (GWAS).**
